# Dynamic plasticity of the lipid antigen-binding site of CD1d is crucially favoured by acidic pH and helper proteins

**DOI:** 10.1038/s41598-020-62833-y

**Published:** 2020-03-31

**Authors:** Bruno Cuevas-Zuviría, Marina Mínguez-Toral, Araceli Díaz-Perales, María Garrido-Arandia, Luis F. Pacios

**Affiliations:** 1 Centro de Biotecnología y Genómica de Plantas (CBGP, UPM-INIA), Universidad Politécnica de Madrid (UPM) - Instituto Nacional de Investigación y Tecnología Agraria y Alimentaria (INIA), Campus de Montegancedo-UPM, 28223 Pozuelo de Alarcón, Madrid Spain; 2 Departamento de Biotecnología-Biología Vegetal, Escuela Técnica Superior de Ingeniería Agronómica, Alimentaria y de Biosistemas (ETSIAAB), Universidad Politécnica de Madrid (UPM), 28040 Madrid, Spain

**Keywords:** Computational biophysics, Molecular modelling

## Abstract

CD1 molecules present lipid antigens for recognition by T-cell receptors (TCRs). Although a reasonably detailed picture of the CD1-lipid-TCR interaction exists, the initial steps regarding lipid loading onto and exchange between CD1 proteins remain elusive. The hydrophobic nature of lipids and the fact that CD1 molecules are unable to extract lipids from membranes raise the need for the assistance of helper proteins in lipid trafficking. However, the experimental study of this traffic in the endosomal compartments at which it occurs is so challenging that computational studies can help provide mechanistic insight into the associated processes. Here we present a multifaceted computational approach to obtain dynamic structural data on the human CD1d isotype. Conformational dynamics analysis shows an intrinsic flexibility associated with the protein architecture. Electrostatic properties together with molecular dynamics results for CD1d complexes with several lipids and helper proteins unravel the high dynamic plasticity of the antigen-binding site that is crucially favoured by acidic pH and the presence of helper proteins.

## Introduction

Adaptive immune responses critically depend on interactions between T-cell receptors (TCRs) and antigen-presenting molecules on the surface of antigen-presenting cells. In mammals, peptide-presenting major histocompatibility complex (MHC) classes I and II and lipid-presenting cluster of differentiation 1 (CD1) are the main antigen-presenting molecules. The generation and loading of peptides onto MHC-I and MHC-II proteins and the molecular mechanisms associated with MHC-mediated responses are well understood^1–4^. However, the knowledge of lipid antigen presentation is far less complete. Since the essential review by Barral and Brenner in ^5^, considerable progress in the understanding of the processing and presentation of lipid antigens has been achieved^6–8^. In particular, the mechanisms of T-cell activation through CD1-lipid-TCR interactions have emerged and even though important gaps remain, a detailed picture of lipid antigen display to TCRs is available^1,6–8^. However, the initial steps regarding the loading onto and exchange between CD1 proteins remain elusive.

**Figure 1:**
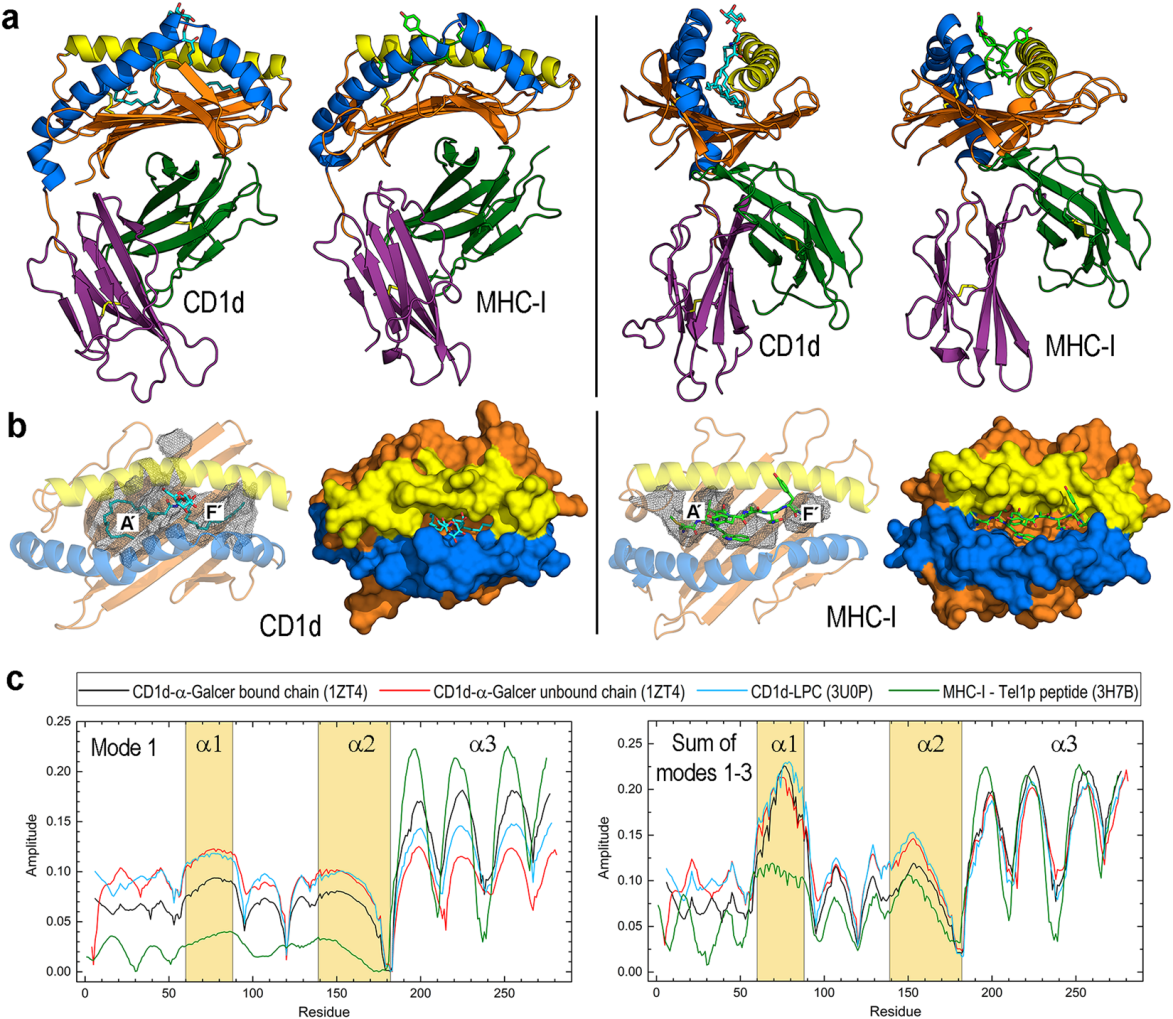
Structure and lowest frequencies normal modes of human CD1d and MHC-I. (**a**) Two views of CD1d in complex with α-GalCer (sticks with carbons in cyan, PDB id 1ZT4) and MHC-I in complex with the Tel1p peptide (sticks with carbons in green, PDB id 3H7B). Right views are obtained upon a 90° rotation of left views around a vertical axis. The domains α1/α2 are coloured in orange with portal helices α1 and α2 in yellow and blue, respectively. The domains α3 and β2 m are coloured in deep violet and dark green, respectively. (**b**) Pockets (gray meshes) identified with DoGSite and molecular surfaces of the domains α1/α2 of the CD1d and MHC-I complexes shown in (**a**). Yellow and blue surface patches correspond to the portal helices α1 and α2, respectively. (**c**) Plots of mode 1 and sum of modes 1–3 of α chains in: (*i*) ligand-bound and (*ii*) unbound forms of the CD1d-α-GalCer complex (1ZT4), (*iii*) CD1d-LPC complex (3U0P), and (*iv*) MHC-I-Tel1p peptide (3H7B) complex. Sequence segments defining helices α1 and α2 are shadowed in light orange. The domain α3 begins at residue 187 in CD1d and at residue 185 in MHC-I.

CD1 molecules are MHC-I-like glycoproteins organised early according to sequence homology into two groups: group 1 is composed of CD1a, CD1b, CD1c, and CD1e and group 2 is composed of the single member CD1d^9^. Of these five CD1 isotypes, a-d are transmembrane proteins that present lipid antigens to TCRs at the cell surface, while CD1e remains intracellular, is the only CD1 member that exists in soluble form and can apparently function as a lipid transfer protein (LTP)^10^. Mammals express CD1 molecules, humans express the five isoforms, and only CD1d is expressed in humans and mice. The common antigen-presenting function of MHC-I and CD1 proteins underlies their similar structure and the existence of antigen-binding clefts (Fig. 1a), cellular pathways and overall modes of TCR interaction. However, the distinct physicochemical nature of peptides and lipids gives rise to important differences in their processing. MHC molecules are highly polymorphic, while CD1 molecules are non-polymorphic. There are distinct modes of MHC and CD1 gene regulation, and CD1 proteins, compared with MHC, have more simplified population genetics^4,7,8^. Moreover, using normal mode analysis (NMA), we show here that despite their structural similarity, the architecture of CD1d presents essential differences in its conformational dynamics with respect to that of MHC-I.

CD1 molecules are highly versatile in presenting lipid antigens. Differences among isotypes in lipid specificity result from variability in the shape and size of hydrophobic channels and portal entrances and from cellular localisation, recycling pathways and exposure to distinct pools of lipids. CD1 isoforms are thus able to discriminate lengths of aliphatic chains and types of polar headgroups which allows them to bind a large variety of lipids^5–8^. Crystal structures of apo- and holo-forms of CD1 isotypes in complex with diverse lipid antigens have been paramount in providing insight into lipid binding details. As of March 14^th^ 2020, a PDB search gave 9 entries for CD1a, 19 for CD1b, 6 for CD1c, 115 for CD1d (including 66 complexes with TCR), and just 1 for CD1e^10^. While the structures of the a,b,c,and e isoforms are from humans, those of CD1d are from humans and mice. Lipid-binding details of isoforms a-c may be found in recent references for CD1a^11^, CD1b^12^ and CD1c^13^. On the other hand, the specificity of TCRs of invariant natural killer T (iNKT) cells in recognising self- or foreign lipids presented solely by CD1d and the key role played by those cells in innate and adaptive immune responses to infection, allergy, autoimmune disease and cancer^14^ explains the amount of structural and biochemical data collected on CD1d. The fact that CD1d is also expressed in mice has helped to examine CD1d-lipid-TCR interactions^15,16^ in greater depth than in any other isotype

Despite this progress in understanding CD1-mediated antigen processing, little is still known about the mechanistic details of lipid loading onto CD1 proteins. This loading needs the assistance of helper LTPs, as CD1 molecules are unable to extract lipids from membranes. The transport of dietary and self-lipids involves the assembly of apolipoprotein-B (apoB)-containing very low-density lipoproteins (VLDL) in the endoplasmic reticulum (ER). This assembly requires assistance from the ER-resident microsomal triglyceride transfer protein (MTP) which also facilitates loading of endogenous lipids onto CD1d in the ER^5,17^. Very recently, the crystal structure of human MTP has provided essential details to understand its poorly characterised mechanisms of action^18^. MTP is a heterodimer consisting of a large α-subunit and a disulfide isomerase β-subunit that acts as a chaperone to stabilise nascent apoB molecules. The lipid-binding and transfer functions of MTP are played by its C-terminal domain which harbours a narrow lipid-binding cavity^18^. Upon assembly in the ER, the complexes of self-lipids with CD1 molecules follow the secretory route to the plasma membrane and are then internalised into early or sorting endosomes. This trafficking is controlled by specific interactions of tyrosine-based targeting sorting motifs in the cytoplasmic tails of CD1 molecules with adaptor proteins AP2 and AP3. CD1 proteins can subsequently follow two routes from the sorting endosome: CD1a and Cd1c move back to the plasma membrane while CD1b and CD1d traffic to late endosomes and lysosomes where they can exchange their loaded self-lipids with other endogenous or foreign lipids^5,17^.

The extraction of bound lipids from CD1 molecules and the loading of other antigenic lipids are facilitated by LTPs in late endosomes and lysosomes. Within the small group of LTPs localised in the endosomal pathway, ganglioside monosialic acid 2 activator protein (GM2AP), Niemann-Pick C2, and saposins A-D have been identified^5,19^. In addition, CD1e is known to mediate lipid exchange with CD1b and could also function as an LTP^10^. On the basis of results showing that saposins and GM2AP are also able to unload lipids bound to CD1d, a “tug-of-war” model in which LTPs and CD1d compete for the same lipid antigen was proposed in^20^. This model suggests that lipid exchange between CD1 and LTP would occur according to their respective affinities for lipids through the formation of transient CD1d-lipid-LTP complexes^20^. There are still no structural data supporting the “tug-of-war” model.

Peptide trafficking and loading onto MHC-I and MHC-II molecules present some similarity with lipid trafficking and loading onto CD1 molecules in that helper proteins are also needed, but there is a great difference in the nature of this assistance. Both proteins MHC-I and MHC-II present peptides at the cell surface to CD8+ and CD4+ T-cells, respectively. MHC-I presents intracellular antigenic peptides generated by degradation of proteins in the proteasome. In order to access MHC-I molecules, those peptides are moved to the ER by transporters associated with antigen presentation (TAP) protein that also assists in the folding of MHC-I. The dedicated chaperone tapasin interacts with TAP, thus coupling the transport of peptides into the ER with their delivery to MHC-I. Upon peptide binding, the chaperones are released, and the assembled peptide-MHC-I complexes leave the ER for antigen presentation at the cell surface. MHC-II molecules present exogenous peptides and protein fragments generated at distinct cellular locations, assemble in the ER and associate with the invariant chain Ii. The Ii-MHC-II complex is transported to the MHC-II compartment (MIIC) where the endocytosed proteins and Ii are degraded, leaving a class II-associated Ii peptide (CLIP) that remains in the peptide-binding groove of MHC-II. The dedicated chaperone HLA-DM (H2-MD in mice) facilitates the exchange of the CLIP for a specific antigenic peptide derived from the protein degraded. MHC-II-peptide complexes are then transported to the plasma membrane to present the antigenic peptide to CD4+ T-cells^4^. Hence, while similar to the abovementioned LTPs that aid in lipid-loading onto CD1 molecules, tapasin and HLA-DM assist in peptide editing and loading onto MHC-I and MHC-II molecules respectively. However, unlike LTPs, tapasin and HLA-DM play the role of a chaperone during the assembly of MHC-peptide complexes. This essential functional difference is reflected in the structural features of tapasin^21–23^ and HLA-DM^24,25^, which bear no resemblance to LTPs in either their size or architecture.

In contrast with assays that study peptide-MHC systems, the hydrophobic nature of lipids raises considerable challenges for investigating the biology of lipid loading onto CD1 proteins. Moreover, the fact that lipids are usually associated with membranes makes the experimental study of lipid loading in late endosomal and lysosomal compartments extremely difficult. Lipid trafficking in endosomal vesicles poses problems that can be addressed by computational approaches to obtain structural data intended to provide mechanistic insight. While crystal structures have been crucial to understanding CD1-antigen interactions, they reveal a static picture of the end result of lipid recognition, and although indispensable for addressing recognition mechanisms, they only describe a snapshot of the interactions. Furthermore, crystal packing can impose non-realistic conditions that are absent in biological environments. In this regard, molecular dynamics (MD) is a valuable computational tool to probe the dynamic evolution of biomolecular systems, providing a time-dependent picture that emerges from interatomic interactions and allows the consideration of external effects (pH, presence of ligands or other proteins, etc.). All-atom MD simulations were used to study the five human CD1 isoforms in their lipid-free and lipid-bound states^26^. This study reported MD results obtained with several force fields and programs for antigen-free CD1 proteins and single 100 ns simulations for a unique holo-form of each CD1 isotype taken from crystal structures^26^. MD was also used to explore distinct states of CD1c in its apo form and two complexes with stearic acid and different small ligands^13^. However, neither of these MD studies addressed the effects arising from the low pH existing in endosomes/lysosomes, the presence of helper LTPs or different lipid antigens on the same CD1 isotype.

Here, we present a computational study of CD1d complexes with different lipids: α-GalCer (α-galactosylceramide known to induce type I iNKT-cell activation^5^), LPC (lysophosphatidylcholine presented by CD1d and recognised by a native, LPC-specific iNKT TCR^27^), phytosphingosine (PHS) and the natural ligand of Pru p 3 (shortened to ligPp3 hereafter)^28^. Experimental structures exist only for α-GalCer and LPC complexes. We also addressed complexes of CD1d with GM2AP, saposin A (sapA) and Pru p 3 proteins. All these systems were studied at pH 7 and 4.5, a value representative of acidic media in late ensodomes/lysosomes. Our work involves the calculation of molecular properties, Poisson-Boltzmann electrostatic potentials (PBEPs), electric fields as well as all-atom MD simulations. The reason for including the peach allergen Pru p 3 (prototype of plant nonspecific LTPs, the most prevalent food allergen in Mediterranean countries and a model of food allergy^29,30^) is our recent experimental report showing that binding of a natural lipid ligand^28^ to Pru p 3 provokes allergic sensitisation by CD1d-mediated activation of iNKT cells^31^. This ligand is composed of 10-hydroxy-camptothecin linked to a lipidic PHS tail^28^ and is presented by CD1d to iNKT cells acting as an adjuvant to promote IgE sensitization to Pru p 3^31^. Of note, one tail of α-GalCer is PHS, known to be an important ligand of CD1d^32^. Our results show dramatic differences in the electrostatic nature of CD1d that dynamically affect the volume and aperture of its antigen-binding cavity occurring either in holo-forms or in the presence of helper proteins only at pH 4.5.

## Results

### Significant differences in antigen-binding grooves and conformational dynamics are associated with similar structures in CD1d and MHC-I

Human CD1d and MHC-I exhibit a similar architecture composed of two chains: an α chain comprised of α1, α2 and α3 domains and a non-covalently linked chain consisting of a β2-microglobulin (β2 m) domain (Fig. 1a). The antigen-binding domain (hereinafter referred to as α1/α2) sits above a 6-stranded β-sheet platform and consists of two antiparallel portal helices, α1 and α2, that flank the entrance to a cavity formed by hydrophobic channels. Although these channels resemble the peptide-binding groove of MHC-I molecules and are also classified as A′ and F′, CD1 proteins have a deeper and narrower ligand-binding compartment (Fig. 1a,b). The α1/α2 domain is linked to an Ig-like α3 domain attached to membranes through a transmembrane segment (not shown in Fig. 1). The β2 m domain is a β-sandwich fold stabilised by a disulfide bridge and is necessary for functional expression of the protein complex on the cell surface.

The crystal structure of human CD1d complexed with α-GalCer (PDB id 1ZT4)^33^ has two molecules per crystallographic asymmetric unit, but only one unit contains the ligand. The comparison of lipid-bound and unbound chains enabled the identification of two different conformations thus providing clues on the ligand-binding details^33^. The lipid fits tightly in the cavity, with the 18 C-PHS tail of α-GalCer bound in the F′ pocket, the 26 C-acyl chain harboured in the A′ channel^33^ and the galactose moiety exposed at the entrance portal (Fig. 1b). In contrast, the binding site in MHC-I is a wide, open groove in which the peptide remains exposed (the much smaller pocket detected by DoGSite in MHC-I compared with that of CD1d is a consequence of the groove of MHC-I being mostly exposed so that the searching algorithm goes “deeper” into the cavity surface to find pockets). This essential difference in the antigen-binding site could be viewed as the result of a greater distance between portal helices α1 and α2 in MHC-I than in CD1d (Fig. 1a,b).

NMA is a computational technique to explore the flexible states of a protein when it moves about equilibrium positions. These states usually have functional significance, and thus, a NMA is a computationally affordable way to probe protein flexibility in order to infer functional information from the structure. NMA is based on basic physics to treat small oscillations: when a vibrating system is perturbed, a restoring force acts to restore the system back to equilibrium. This problem is solved in mechanics by constructing a matrix formed by the second derivatives of the potential energy of the system with respect to the spatial displacements and then diagonalising this matrix to obtain a set of eigenvectors and eigenvalues. If the system is a molecule, the eigenvectors give the spatial displacements of all atoms that define the vibrational modes of the molecule and the eigenvalues give the squares of the frequencies of these vibrations. The vibrational modes involve the independent motions of atoms so that the low frequency modes in proteins describe large-scale conformational changes. In contrast with computationally expensive MD simulations, NMA is a technique that at only a small fraction of the cost is able to provide valuable information on the possible conformational changes of a protein and extract this information from a single equilibrium structure. Although NMA provides a far less complete dynamic description of a protein, it permits us to sample conformational dynamics upon examining the backbone of a structure alone. The reader can find an updated review on NMA of proteins in ref. ^34^. and the references therein.

Despite the structural similarity between the proteins MHC-I and CD1d, an NMA to sample conformational dynamics of the α chain performed on the complete structure (i.e., in the presence of β2 m although its modes are not shown in Fig. 1c; plots in this figure show the amplitude of each mode over the sequence), reveals differences along the complete chains. We also extended NMA to the unbound chain of the CD1d-α-GalCer complex and to the human CD1d-LPC complex (PDB id 3U0P)^27^ (Fig. 1c). It has long been known that lowest-frequency modes can be associated with fundamental characteristics of proteins, mostly related to their function^34,35^. Since modes are ranked by increasing frequency, mode 1 is the lowest-frequency mode. For the systems studied here, mode 1 has a greater amplitude at the α1/α2 domain in CD1d complexes than MHC-I and the opposite occurs at the α3 domain, suggesting that the α1/α2 domain has a slightly greater flexibility in CD1d than in MHC-I regardless of the rather similar architectures. While mode 1 profiles display a similar pattern in all CD1d forms, it shows a greater amplitude at the α1/α2 domain in the unbound chain than in the bound chain of the α-GalCer complex, with this mode in the LPC complex being more similar to the unbound chain (Fig. 1c). Of note, the sum of modes 1-3 (the three lowest frequency modes and therefore the modes with possible larger scale conformational changes) shows a significantly greater amplitude just at portal helix α of the three chains of CD1d than that in MHC-I, which displays a plateau at the α1 segment, whereas profiles of this sum are very similar at the remaining regions in all cases (Fig. 1c).

### Acidic pH has a significant impact on the electrostatic nature of the CD1d antigen-binding domain

**Figure 2:**
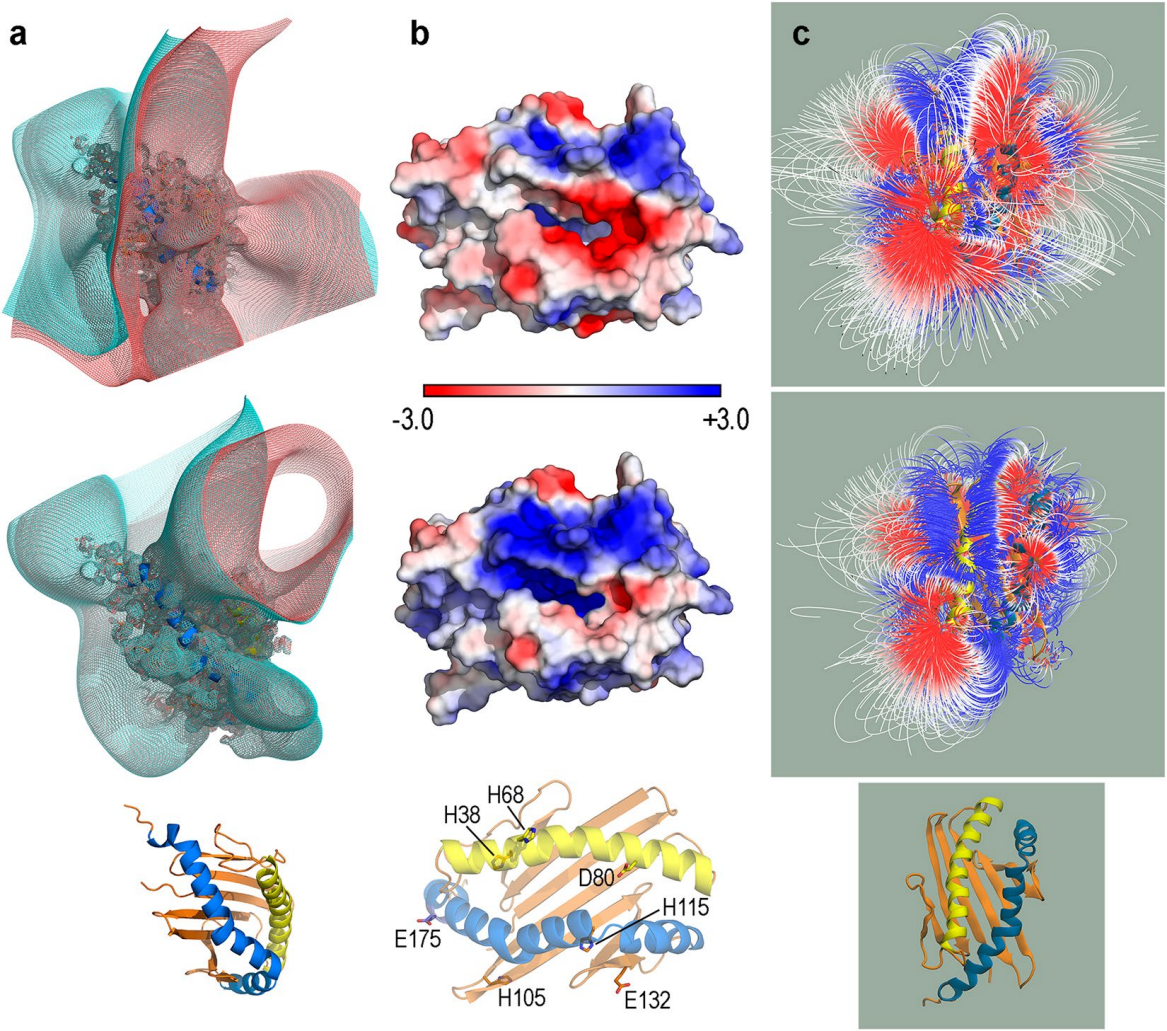
Poisson-Bolztmann electrostatic potential (PBEP) of the domain α1/α2 of the ligand-bound chain of human CD1d in complex with α-GalCer (PDB id 1ZT4). (**a**) Mesh isosurfaces +0.001 (aquamarine) and −0.001 (salmon) of PBEP at pH 7 (top row) and 4.5 (middle row) drawn at the orientation shown at the bottom of the column. (**b**) PBEP mapped onto the molecular surface (scale bar in *kT*/*e* units) at pH 7 (top row) and 4.5 (middle row) drawn at the orientation shown at the bottom of the column, where the 7 residues that are protonated at pH 4.5 are labelled. (**c**) Electric field created by the PBEP (gradient magnitude −2.0 in red to +2.0 in blue) at pH 7 (top row) and 4.5 (middle row) drawn at the orientation shown at the bottom of the column.

The α1/α2 domain extending from the N-terminus up to residue 186 in the crystal structure of the human CD1d-α-GalCer complex^33^ has 7 Asp + 10 Glu and 9 Lys + 9 Arg residues, which yields a net charge +1 at pH 7. At pH 4.5, a value representative of the acidic media in late endosome/lysosome compartments, 1 Asp + 2 Glu together with 4 His in this domain are protonated now yielding a net charge +8. PBEP calculations on the ligand-bound chain in this complex reveal dramatic differences in the electrostatic nature of the space around the entrance portal (Fig. 2). PBEP zero isocontours (bracketed by ±0.001 isocontours for visualisation purposes in Fig. 2a) are completely different in this space, changing from negative at pH 7 to positive at 4.5. In addition, the negative/positive boundary defines a large open space above the portal at pH 7 but a convoluted shape at pH 4.5, leaving a positive large groove between helices α1 and α2 (Fig. 2a). The PBEP mapped onto the protein surface also displays a sharp difference between both pH values just in the immediate vicinity of the portal entrance that changes from clearly negative at pH 7 to strongly positive at pH 4.5. Of note, among the 7 residues protonated at pH 4.5, only His68, Asp80 (α1) and Glu175 (α2) are located in portal helices although the glutamate is far from the portal entrance (Fig. 2b). PBEP thus suggests that protonation of His68 and Asp80 is paramount in providing a strongly positive electrostatic nature to the lipid binding groove. Field lines of electric field ***E*** created by the PBEP *V*(**r**), ***E*** = −∇*V*(**r**) reinforce those changes (Fig. 2c). In fact, field lines originating at positive PBEPs are confined to small regions outside the portal entrance, while they form a sharply defined groove running all the way along the space between helices α1 and α2 (Fig. 2c).

### The lipid-binding cavity in CD1d displays high dynamic plasticity arising from lipid-dependent features

**Figure 3:**
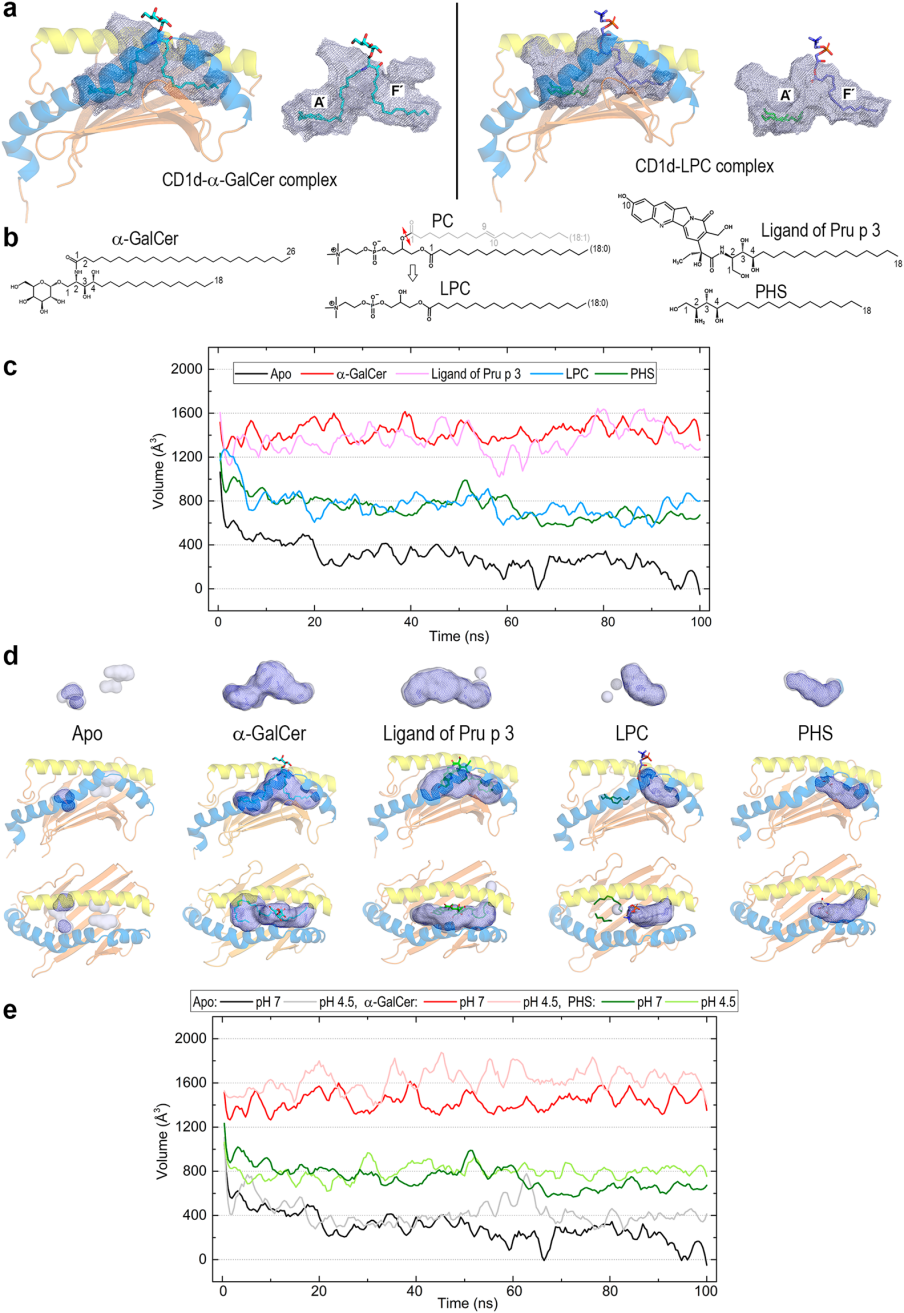
Antigen-binding pockets in human CD1d. (**a**) Cavities computed with DoGSite in the crystal structures of CD1d in complex with α-GalCer (sticks with carbons in cyan, PDB id 1ZT4) and with LPC (sticks with carbons in deep blue; C11 and C6 spacer chains identified in the electron density are shown as sticks with carbons in green, PDB id 3U0P). (**b**) Lipid ligands in the CD1d complexes studied in this work. LPC is the result from hydrolysis of phosphatidylcholine (PC) at the bond marked with a double red arrow. PHS is present in both α-GalCer and ligPp3. (**c**) Variation along 100 ns all-atom MD simulations of the cavity volumes in the apo-form of CD1d and in its complexes with α-GalCer, ligPp3, LPC and PHS at pH 7 (plots smoothed with Savitzky-Golay filtering). (**d**) Cavity shapes defined by pocket occupancy between 0.6 and 0.8 (bluewhite transparent surfaces) and between 0.8 and 1.0 (deep blue meshes) along the simulations except the apo-form for which the surface and the mesh correspond to 0.4–0.6 and 0.6–0.8 occupancies, respectively (no 0.8–1.0 occupancy detected). (**e**) Variation along 100 ns all-atom MD simulations of the cavity volumes of apo-CD1d and of CD1d in complex with α-GalCer and PHS at pH 7 and 4.5 (plots smoothed with Savitzky-Golay filtering). Data in (**c**–**e**) computed with PocketAnalyzer.

Crystal structures of human CD1d complexed with α-GalCer^33^ and LPC^27^ show a large cavity with both the A′ and F′ channels occupied by ligands (Fig. 3a). While the former structure has the two tails of α-GalCer (Fig. 3b) filling both channels (26C in A′, 18C in F′), thus being most probably an “optimum” case of channel occupancy, the latter structure has the single chain of LPC in the F’ pocket and two spacer C11 and C6 hydrocarbon chains identified in the A’channel^27^ (Fig. 3a). Together with these two crystal structures, we addressed complexes without experimental structures with two lipids that induce iNKT cell activation: ligPp3 identified as 10-OH-camptothecin-PHS^28^ and PHS alone, which is also the 18C tail of α-GalCer (Fig. 3b). CD1d is known to bind PHS^32^, and it has also been suggested that iNKT activation in humans should contain a PHS base^36^.

MD simulations on apo- and holo-forms of CD1d show clear trends regarding the antigen-binding cavity (Fig. 3c,d). As has been recognised^26,33^, although the non-lipid binding chain adopts an “open” conformation in 1ZT4 compared with the lipid-binding chain (Table 1)^33^, its dynamics lead to “collapse” of the cavity^26^. In fact, our MD results show that the initial volume of apo-CD1d falls at the very beginning of the simulation and decreases over time to the extent that the cavity shrinks, leaving only two tiny enclosures with low occupancy during simulation (Fig. 3c,d). In contrast, the cavity volumes in both the α-GalCer and ligPp3 complexes remain at large values with large shapes associated with 0.8–1.0 occupancy during simulation. The cases of LPC and PHS are intermediate in that both CD1d complexes show slightly decreasing volumes with values overall smaller than the preceding complexes. Shapes associated with high 0.8–1.0 occupancy are large enough only at channel F′ occupied by the single aliphatic chain of these two lipids. As far as dynamic variation of cavity volumes is concerned, pH has no significant effect (Fig. 3e) because the dramatic electrostatic changes occurring in acidic media presented above affect external regions. In summary, these MD results suggest that the antigen-binding cavity of CD1d is a plastic enclosure that dynamically adapts to the lipid antigen. In other words, this plasticity arises from the nature of aliphatic chains harboured in inner channels. Note that all plots in Fig. 3 are relatively flat, i.e., the cavity volumes change steadily without excessive fluctuations, a sharp contrast to that found for an apparently related geometric property such as the aperture of the groove portal, which is addressed next.

**Table 1:** Aperture of the groove portal defined by the distance between alpha carbons of residues in helices α1 and α2 flanking the entrance to the portal in the crystal structures of complexes of CD1 isotypes and average values computed in 100 ns all-atom MD simulations for the CD1d systems.

Isotype	Crystal structure	Residues α1 - α2	Distance (Å)
CD1a	LPC complex, 4X6E^11^	S77–N151	13.2
CD1b	Phosphatidylserine complex, 5WKE^12^	F77–Y151	13.4
CD1c_1	PC complex, 6C15^37^	L77–Y152	11.4
CD1c_2	Complex with cholesteryl esters, 5C9J^13^	D80–Y155	11.5
CD1d_bound	α-GalCer complex, bound chain 1ZT4^33^	F77–D151	13.1
CD1d_unbound	α-GalCer complex, unbound chain 1ZT4^33^	F77–D151	14.3
CD1e	Uncomplexed, 3S6C^10^	F73–Y144	14.8
**Average F77 – D151 distance and standard deviation (Å) in CD1d obtained in 100 ns MD simulations**			
**CD1d system**	**Av. Distance**	**Std. dev**.	
Apo-form at pH 7	11.9	0.70	
Apo-form at pH 4.5	15.7	1.97	
α-GalCer complex at pH 7	13.6	0.56	
α-GalCer complex at pH 4.5	13.6	0.44	
LigPp3 complex at pH 7	15.2	1.03	
LPC complex at pH 7	14.2	0.59	
PHS complex at pH 7	14.6	1.14	

**Figure 4:**
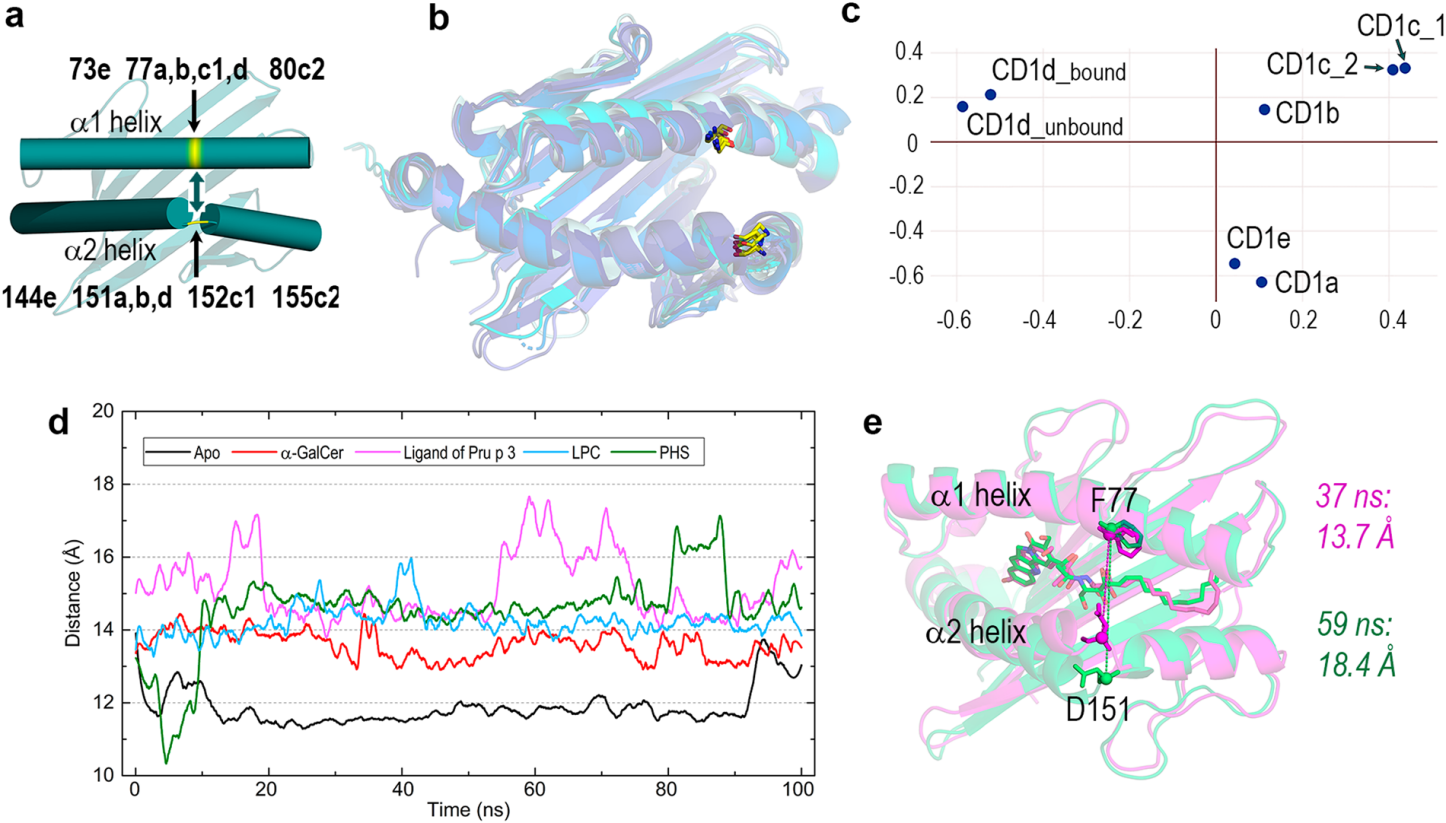
Aperture of the groove portal and multiple structural alignment computed with DALI of the domains α1/α2 of the crystal structures of human CD1 isoforms in Table 1. (**a**) Location in helices α1 and α2 of residues flanking the entrance to the portal used to measure the aperture of the groove portal as the distance between their alpha carbons. In the helix α1, these residues are: 73 in CD1e, 77 in a,b c1 and d isoforms, and 80 in CD1c2. In the helix α2, these residues are: 144 in CD1e, 151 in a,b and d isoforms, 152 in CD1d and 155 in CD1c2. Values of those distances are given in Table 1. (**b**) Superposition of the seven structures in Table 1. CD1 cartoons are colored in blue hues from deep blue for CD1a to light cyan for CD1e. Backbone atoms of residues indicated in (**a**) are shown as sticks with carbons in yellow (**c**) Multidimensional scaling correspondence analysis performed by DALI with most similar structures positioned near each other. (**d**) Variation of the Cα.F77-Cα.D151 distance in the apo-form of CD1d and in its complexes with α-GalCer, ligPp3, LPC and PHS at pH 7 (plots smoothed with Savitzky-Golay filtering). (**e**) Comparison of two structures for the ligPp3 complex at two frames in the trajectory that show very different values of the Cα.F77-Cα.D151 distance.

The distance between the α-carbons of residues flanking the entrance defined by portal helices at the hinge of the α2 helix (Fig. 4a) has been proposed as a measure of its aperture^10^. This distance varies in crystal structures of complexes of the five CD1 isotypes between ~11.5 Å for two recent CD1c complexes with cholesteryl esters^13^ and with PC^37^ to 14.8 Å for uncomplexed CD1e^10^ (Table 1). The mentioned presence in the CD1d-α-GalCer crystal of lipid-unbound and bound chains allows us to assess the effect on this aperture of the conformational change provoked by the lipid: 14.3 and 13.1 Å, respectively (Table 1). However, all these values are considerably shorter than typical analogous distances of approximately 18–20 Å in MHC I and II molecules^38^. The multiple superposition of these seven CD1 chains (besides both CD1d chains, the two mentioned complexes of CD1c are included as double checks of their anomalous short distance) reveals a nearly coincident location of backbone atoms of the residue in helix α1 and a slightly larger variance in those of the residue in helix α2 (Fig. 4b). Structural similarity scores in this superposition define three clusters: (i) CD1b and both CD1c chains, (ii) CD1a and CD1e, and (iii) both CD1d chains (Fig. 4c). Isotypes with rather different portal apertures are thus clustered together.

In contrast with the results found in cavity volumes, MD results show less clear trends for this portal distance. At pH 7, the α-GalCer and LPC complexes display relatively flat curves with small deviations, whereas ligPp3 and PHS complexes show longer distances with large standard deviations (Fig. 4d and Table 1). Comparing structures at two frames of ligPp3 trajectory for which the portal distance exhibits rather different values, it can be seen that in agreement with the results stated in the preceding paragraph, the backbone at α1 F77 remains unaltered while that of α2 D151 shifts outward, thus opening the entrance (Fig. 4e). Both ligPp3 and PH3 complexes present occasional fluctuations of their OH groups, which are exposed at the portal region. The apo-CD1d form in our MD calculations was the lipid-unbound chain of its α-GalCer complex in the crystal structure 1ZT4, at which this distance is 14.3 Å. MD results reveal that this distance rapidly decreases and stabilises at ~11.9 Å with small deviation (Fig. 4d and Table 1), which is in agreement with the aforementioned cavity closure in the absence of lipids. To assess pH effects on this aperture, we performed further simulations at pH 4.5 for both lipid-bound and unbound chains of 1ZT4 (Supp. Fig. 1). MD results indicate that the stabilisation provided by α-GalCer filling both the A’ and F’ channels produces a constant distance of ~13.6 Å in the bound chain with low standard deviations at both pH values. In sharp contrast, the apo form has an average distance of 15.7 Å with an enormous deviation at pH 4.5 (Table 1). This distance also shows a rapid initial decrease but after 10 ns, this distance starts to increase with very large oscillations and then stabilises at approximately 60 ns at >16 Å (Supp. Fig. 1).

We also computed the bending of the portal helices over the MD simulations in these CD1d systems (Supp. Fig. 2). The major result is that α1 suffers greater changes than α2, deviating ~30° from linearity when lipid chains occupy both channels, whereas α1 remains nearly straight in apo-CD1d. In contrast, the two segments of α2 that are bent at ~90° in some complexes show slightly larger angles of up to 100° without suggesting any lipid-specific features (Supp. Fig. 2).

### MD calculations suggest that helper GM2AP affects CD1d only at acidic pH

**Figure 5:**
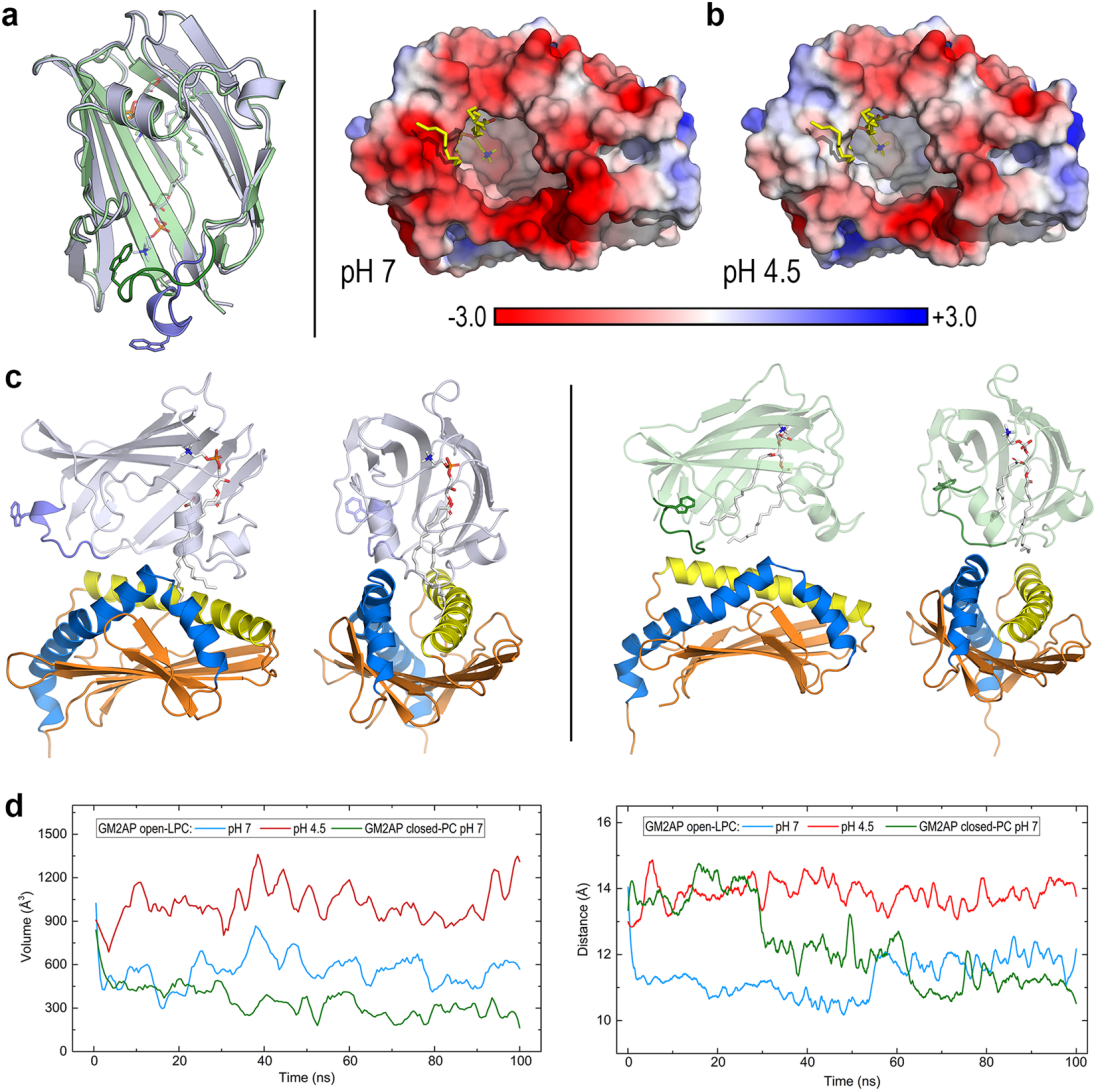
CD1d-GM2AP complex. (**a**) Crystal structure of the GM2AP-PC complex (PDB id 2AG2). Superposition of the open (light blue, chain A in 2AG2) and closed (light green, chain C in 2AG2) conformations of GM2AP. The backbone of the flexible loop 127–135 and the side chain of W133 are coloured in deep blue (open) and deep green (closed). The PC ligand in the internal cavity is shown as sticks. (**b**) PBEP computed at pH 7 and pH 4.5 mapped onto the protein surface of the open conformation of GM2AP in complex with oleic acid (OLA) and LPC (PDB id 2AG4, ligands shown as sticks with carbons in yellow) used in MD simulations of the CD1d-GM2AP (open) system. (**c**) Left panel: Front (left) and side (right) views of the initial geometry of the CD1d-GM2AP (open)-OLA-LPC complex. Right panel: Front (left) and side (right) views of the initial geometry of the CD1d-GM2AP (closed)-PC complex. (**d**) Change along 100 ns all-atom MD simulations of the cavity volumes of CD1d (left panel) and groove portal distance in CD1d (right panel) in complexes displayed in (**c**) (plots smoothed with Savitzky-Golay filtering).

GM2 activator protein (GM2AP) assists lipid trafficking and loading onto CD1 molecules in the endosomal pathway^19^. Crystal structures of human GM2AP complexed with PC and its deacylation products (LPC and oleic acid, OLA) show open and closed conformations arising from a flexible loop formed by residues 127–135 in which Trp133 acts as the lid of a large cavity (Fig. 5a)^39^. Upon obtaining the protonation state of ionisable side chains at pH 7 and 4.5 of the open GM2AP and CD1d proteins in the initial geometries of their complexes as explained in Methods, we computed the PBEP mapped onto the surface of GM2AP (Fig. 5b). The strongly negative potential around the cavity at pH 7 largely disappears at 4.5 as a consequence of protonation of 2 glutamate residues and 3 histidine residues, which changes the net charge of GM2AP from −7 to −2.

MD simulations of CD1d in complex with open and closed conformations of GM2AP and ligands in their crystal structures (LPC + OLA and PC, respectively; Fig. 5c) show that the complex with closed GM2AP dissociates at the very beginning of the simulation (Supp. Fig. 3a) so that CD1d displays dynamic behaviour as if it were isolated. In fact, its RMSD shows a stable pattern (Supp. Fig. 3b) and cavity volume and the aperture of the groove portal exhibit features similar to those of apo-CD1d: collapse of the cavity and decrease of the aperture (Fig. 5d). The complex of CD1d with open GM2AP is stable at both pH values (Supp. Fig. 3a), but there are significant differences in the CD1d portal. At pH 7, the cavity volume decreases rapidly and then oscillates around small values, while the portal aperture closes immediately and remains at short lengths (Fig. 5d). In sharp contrast, both cavity volume and aperture show no decrease and remain stationary (average values of 1018 Å^3^ and 13.9 Å, respectively) at pH 4.5. The bending of portal helices α1 and α2 shows no distinctive features. Helix α1 deviates no more than 10° from linearity irrespective of pH and GM2AP conformation/ligand. Although the angle between the segments of helix α2 starts at values between 105° and 85° at the beginning of the simulations, this angle stabiles near 90° at ~40 ns for all CD1d-GM2AP systems (Supp. Fig. 3c).

### MD calculations suggest a pH-dependent effect of the SapA dimer on the complex geometry and cavity features of CD1d

Initially characterised by their participation in degrading glycosphingolipids in lysosomes, saposins have long been known to bind and release lipids, assisting their loading onto CD1 molecules^5,6,19,20^. However, no direct evidence on the structure of any saposin-CD1 complex exists, the molecular clues that control lipid binding versus release are still unknown, and attempts to measure the interaction of human or murine CD1 molecules with saposins have been unsuccessful so far^19^. This suggests that this interaction could be weak and transient, which in turn would imply a lack of specificity between any saposin and any CD1 isoform^19^. Saposins exist in two states: closed monomeric and open dimeric with a hydrophobic cavity that can harbour lipids and detergents forming lipoprotein discs^40^. In addition, their structural, electrostatic and dynamic features are strongly pH-dependent^41^.

**Figure 6:**
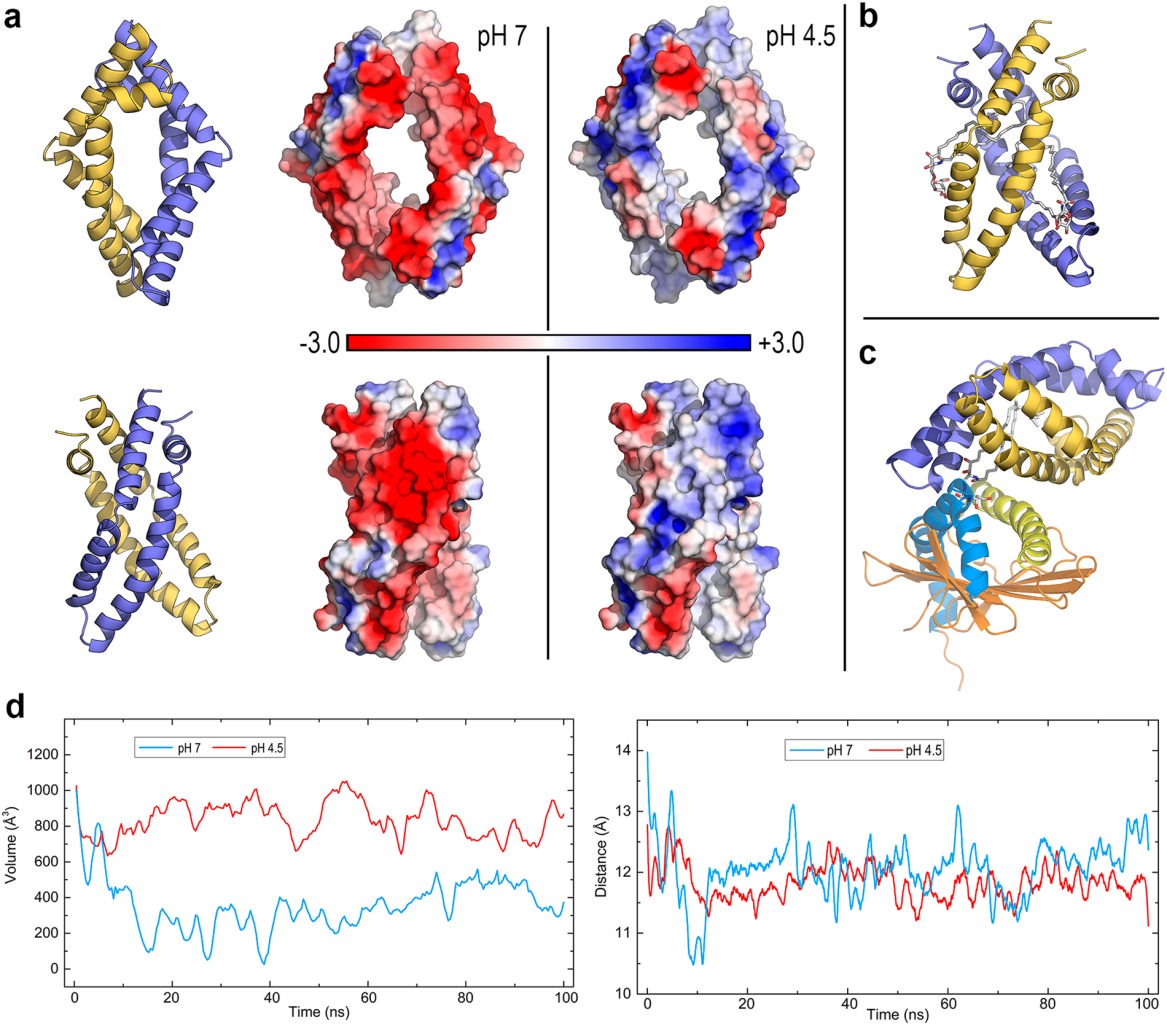
CD1d-SapA dimer complex. (**a**) Two views of the structure of dimeric SapA (open) modelled on the basis of the recent crystal structure of the SapA dimer in complex with GALC (PDB id 5NXB) and PBEP mapped onto the protein surface at pH 7 and pH 4.5 in the orientations shown on the left. (**b**) Optimised structure of the complex of SapA (open) dimer with two molecules of α-GalCer (sticks with carbons in white) initially modelled as explained in the text. (**c**) Initial geometry of the CD1d-SapA(open) dimer-α-GalCer complex. The SapA chain (D) making more contacts with CD1d is coloured in light orange while the other chain (C) is coloured in slate blue. (**d**) Change along 100 ns all-atom MD simulations of the cavity volumes of CD1d (left panel) and groove portal distance in CD1d (right panel) in the complex displayed in (**c**) at pH 7 and pH 4.5 (plots smoothed with Savitzky-Golay filtering).

The recent crystal structure of hydrolase β-galactocerebrosidase (GALC) in complex with the murine SapA dimer (PDB id 5NXB) is the first structure of a protein-saposin interaction^42^. GALC catalyses the removal of galactose from α-GalCer (Fig. 3b) and requires SapA for lipid processing. In this structure, SapA adopts the same open dimeric form as in lipoprotein discs^40^, shaping a hydrophobic cavity in which at least two molecules of α-GalCer can be shielded from water^42^. We used this murine SapA to model our dimeric open form of human SapA (Fig. 6a) and its complex with two molecules of α-GalCer (Fig. 6b). After considerable protein-protein docking (explained in Methods) for this system, we selected the initial geometry of the CD1d-SapA-dimer-α-GalCer complex shown in Fig. 6c to explore its dynamics via 100 ns MD simulations. We obtained the protonation state of ionisable side chains of both CD1d and SapA at pH 7 and 4.5 and computed the PBEP mapped onto the surface of SapA (Fig. 6a). Similar to that found in GM2AP, the strongly negative potential at pH 7 changes to neutral/positive at pH 4.5 as a consequence of protonating 6 acidic residues in chain D and 2 in chain C (see the Fig. 6 legend) that modify the net charge of the SapA dimer from −16 at pH 7 to −8 at 4.5.

**Figure 7:**
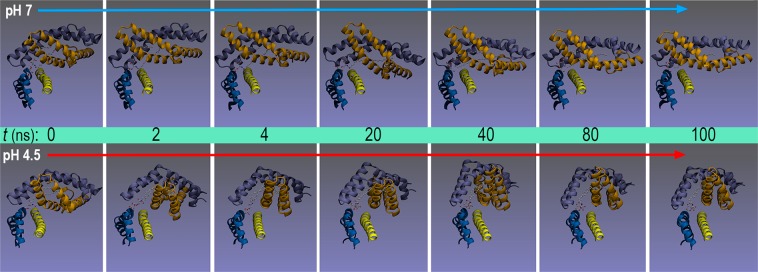
CD1d-SapA dimer complex. Snapshots of the 100 ns all-atom MD simulation at pH 7 and pH 4.5. The two chains of SapA dimer are depicted on top of portal helices α1 (yellow) and α2 (marine blue) of CD1d (the rest of the protein is omitted for ease of visualisation). α-GalCer is shown as ball-and-sticks with carbons in white.

The mobility of open SapA in complex with CD1d is far greater than that in open GM2AP. Even though no dissociation is observed and the RMSD is stabilised, both chains of SapA display large values >10 Å at both pH values, while CD1d shows an RMSD <2 Å (Supp. Fig. 4). For the CD1d cavity and portal features in this complex, a slightly different behaviour to that found with GM2AP is now observed. The cavity volume also decreased rapidly in the first 10 ns, showing small values of ~300 Å^3^ at pH 7, while it remained stable at pH 4.5, although with a smaller average value (855 Å^3^) than that in GM2AP complex (Fig. 6d). However, the portal aperture stabilises at ~12 Å at both pH values, although with much larger oscillations at pH 7 than at 4.5 (Fig. 6d). Interestingly, the main difference in MD results is found in the geometry of partners in this complex. Although the same initial geometry was used for simulations at both pH values, it was observed that the nearly perpendicular arrangement of the SapA dimer and CD1d portal helices holds along the whole trajectory at pH 7, while it starts to change in the first nanosecond and then remains at a nearly parallel orientation between the CD1d portal helices and chain D of SapA, which shifts towards a location on top of the α1 helix, thus orienting the polar head of α-GalCer above the portal entrance (Fig. 7).

With regards to the bending of portal helices, while α1 largely oscillates at pH 7, it remains stabilised at near linearity at pH 4.5, with α2 showing no distinctive features at either pH (Supp. Fig. 4b).

### MD calculations predict no stable CD1d-Pru p 3-ligand complexes

The abovementioned iNKT activation producing sensitisation to the Pru p 3 allergen through CD1d-presentation of ligPp3^31^ led us to address possible complexes via MD simulations. Unlike GM2AP and SapA, this allergen shows no pH-dependent electrostatic changes, as it has a single acidic residue (Asp43 whose pKa <4.0 precludes protonation at pH 4.5) and 4 Lys + 4 Arg yielding a net charge +7 and a rather uniform neutral/positive electrostatic potential (Supp. Fig. 5a). Even though the great majority of protein-protein docking methods predict similar geometries with the allergen on top of the CD1d portal (Supp. Fig. 5b), a number of exploratory 20 ns MD simulations on selected CD1d-Pru p 3 complexes with and without ligPp3 (Supp. Fig. 5c) in the two possible orientations of the allergen with respect to its hydrophobic tunnel^43^ predicted no stable complexes. In fact, while the RMSD remains <3 Å for CD1d, the RMSD is between ~3 and 6 Å for Pru p 3 without the ligand and increases above 10 Å in the presence of ligPp3 irrespective of pH (Supp. Fig. 5d). The cavity volumes and portal distances of CD1d in these complexes decreased as if the protein was in its apo form, revealing no effect from Pru p 3 (Supp. Fig. 5e).

## Discussion

While human proteins CD1d and MHC-I share a virtually identical architecture associated with their common antigen-presenting function, they display subtle differences in their antigen-binding domains that arise upon sampling the conformational dynamics of their backbones via NMA. Lower-frequency modes clearly show greater amplitude in the α1/α2 domain of the lipid-bound chains of CD1d with respect to MHC-I with a particularly large maximum in the region of portal α1 in CD1d that is absent in MHC-I. These NMA results could be rationalised in terms of greater mobility at the α1 (especially) and α2 portal helices arising from the structure of CD1d with respect to MHC-I. It must be stressed that NMA ignores the presence of ligands so that dynamic modes are obtained only from backbone atoms in their complexes.

Protonation of seven residues in the α1/α2 domain of CD1d at acidic pH produces a dramatic change in its electrostatic nature. Decreasing the pH to 4.5 modifies the electrostatic potential in the space around the protein from dominantly negative to overwhelmingly positive and specifically modifies the portal region by turning its strongly negative potential into strongly positive, a change motivated by protonation of two key residues in this region: His68 and Asp80. The effect of acidic pH is thus paramount in electrostatically selecting the type of molecular partners of CD1d. Therefore, both protein-ligand and protein-protein interactions can drastically be altered by protonating only a few residues in CD1d.

The hydrophobic channels that shape the lipid-binding pocket in CD1d show considerable plasticity that suits the aliphatic chains of lipid antigens. Controlled by the space between helices α1 and α2 and by the aperture defined by the distance between two residues flanking the portal entrance in both helices, this cavity varies dynamically to adapt to the lipid harboured within. When unloaded, both the cavity volume and portal aperture of CD1d evolve, decreasing rapidly. Although it has repeatedly been stated that apo-CD1 collapses, it should be stressed that decreasing these cavity features does not involve dynamic destabilisation of the structure. These changes are produced by slight displacements and bendings of helices α1 and α2 that are in turn sensitive not only to the type of loaded lipid (as expected and is actually known) but also to external effects due to pH (i.e., to protonation of key residues) and to the presence of partner proteins. In fact, the MD study of complexes of CD1d with GM2AP and saposin A at pH 7 and 4.5 predicts this result.

While our MD calculations were not intended to address lipid loading, as this process is known to take times far beyond the current limits of MD simulations (μs range)^44^, the results presented here provide insight into the dynamic effects produced on CD1d by partner proteins and acidic pH. In fact, the presence of GM2AP in the open conformation complexed with LPC provokes a cavity volume and portal aperture of CD1d at large values at pH 4.5 as if a lipid antigen is harboured within. In contrast, the same GM2AP-LPC open complex at pH 7 or GM2AP-PC in the closed conformation has no effect on the cavity features of CD1d, which now shows the dynamic evolution characteristics of its apo form, that is, as if there were no external effects. Similar results were found for the CD1d-open SapA-α-GalCer complex: only at pH 4.5 is CD1d observed to keep its volume cavity at large values, although now the only distinctive feature in portal aperture is that its distance oscillates very little, in contrast with the marked oscillations observed at pH 7. Interestingly, this complex displays a nearly parallel orientation of the SapA dimer at pH 4.5 with respect to the groove defined by helices α1 and α2 in CD1d, thus orienting the polar head of α-GalCer above the portal entrance. Notably, similar to CD1d, both GM2AP and SapA drastically change their electrostatic nature at pH 4.5.

Finally, the inclusion of the natural lipid ligand of Pru p 3 in our study obeys the reported experimental evidence that it (i) acts as an adjuvant promoting sensitisation to the allergen through its recognition by CD1d, (ii) is able to interact with iNKT cells upon CD1d presentation and (iii) its immunological activity resides in the PHS tail^31^. However, MD calculations do not predict a stable CD1d-Pru p 3-ligPp3 complex irrespective of pH, suggesting that although the allergen is an LTP, it is unlikely that its ligand would be loaded onto CD1d through a direct interaction with Pru p 3. The presence of ligPp3 or its PHS tail inside CD1d might be explained in terms of its probable location in endosomal membranes and extraction by helper proteins assisting in lipid loading onto CD1d via mechanisms similar to those associated with other lipid antigens.

## Methods

### Structures

The initial structures of the apo- and holo-forms of human CD1d were taken from the two chains in the crystal structure of the human CD1d-α-GalCer complex (PDB id 1ZT4^33^). This entry has two molecules per crystallographic asymmetric unit: the lipid-unbound and lipid-bound chains that were used as initial structures for our apo- and holo-forms of CD1d, respectively, in preparation of the different CD1d systems addressed. For MD simulations, only the α1 + α2 domain of CD1d (residues 6–186 in 1ZT4) was considered.

The crystal structures of human CD1d-LPC complex (PDB id 3U0P)^27^ and human MHC-I in complex with the Tel1p peptide (PDB id 3H7B^45^) were used for NMA. Structures of other CD1 isotypes used for calculating distances to measure portal aperture and for multiple structural alignment analyses as well as those of helper proteins GM2AP and saposin A were taken from several crystal structures that are indicated in the Results subsections in the main text where the proper references are also given.

The initial geometries of the lipid ligands were taken from their complexes with CD1d (α-GalCer) and GM2AP (LPC, PC, and OLA) referenced in the main text except for PHS and ligPp3 whose geometries were taken from the MD final structures of their complexes with Pru p 3^28,31,43^. The initial structure of the peach Pru p 3 allergen was taken from its crystal structure (chain A in PDB id 2ALG^46^).

Multiple structural alignments and scores measuring structural similarity were obtained with DALI^47^ in all-against-all mode (http://ekhidna2.biocenter.helsinki.fi/dali/). For a set of *N* structures, DALI computes the *N*x*N* matrix of pairwise similarities and uses several heuristics to optimise and refine the alignment score. An algorithm for correspondence analysis is applied to obtain a multidimensional scaling projection of the set of *N* structures^48^.

Analysis of structures and calculation and rendering of geometrical features were achieved with VMD 1.9.3^49^ Chimera 1.13^50,51^ and PyMOL 2.3.2 (The PyMOL Molecular Graphics System, version 2.0; Schrödinger, LLC: New York, NY, USA, 2017)

### Normal mode analysis (NMA)

The complete structure (α chain plus β2 m domain) of both the human CD1d and MHC-I proteins was considered in NMA calculations. For CD1d, three different structures were used: the lipid-bound and lipid-unbound chains of the α-GalCer complex (1ZT4) and that of the LPC complex (3U0P). In all cases, the complete structure of each complex was submitted to the DynOmics server (http://gnm.csb.pitt.edu/index.php)^52^ performing Gaussian network model (GNM) calculations. Although the presence of the β2 m domain was taken into account, the 20 lowest frequency normal modes of only α chains were downloaded and processed with in-house scripts to prepare the amplitude plots.

### Calculation and analysis of pockets and cavities

Pocket shapes and volumes for static structures were determined with the DoGSiteScorer service available at https://proteins.plus/. DoGSiteScorer is a grid-based computational method that applies a difference of Gaussian (DoG) filter to detect potential pockets in 3D protein structures^53^. Changes in pocket volumes and occupancy of space in internal cavities along MD trajectories were calculated with PocketAnalyzer^54^. Variation in the distances used to measure the portal aperture in CD1d were computed along MD trajectories with Carma 1.7^55^. Changes along MD trajectories of bending angles in α1 and α2 helices of CD1d were calculated as angles between vectors defined by middle points of distances between α carbons of residues in α1 and α2 indicated in Supp. Fig. 2 with in-house Tcl/Tk scripts running in VMD 1.9.3^49^.

### Calculation of pKa and protonation states

pKa values of ionisable side chains were computed with three different methods: Propka 3.1^56,57^ (available at http://nbcr-222.ucsd.edu/pdb2pqr_2.0.0/), H++^58,59^ (available at http://biophysics.cs.vt.edu/index.php), and Rosetta-pKa protocol^60^ (available at https://rosie.graylab.jhu.edu/pka/). pKa predictors use empirical models to estimate pKa values of the side chains of Asp, Glu, His, Tyr, Lys, and Arg residues by sampling their protonated and deprotonated states through free energy calculations for deprotonation in their local environment in protein structures. As these estimated pKa values may have considerable uncertainties, it is advisable to employ more than one method to try to improve the reliability of the results. In our case, we selected pKa values under the constraint that at least two out of the three methods predicted similar values. For the pH range and protein complexes addressed in this work, the pKa values for Asp and Glu were unambiguously determined in this way, and in the case of His, the three methods agreed to predict rather similar pKa values. Then, the side chains of Asp and Glu were assumed to be protonated (thus having a 0 charge) if their pKa values were ≥pH, and His side chains were protonated if their pKa values are ≤pH (thus having +1 charge). In both cases, a (±0.1 pH units) interval was allowed to account for the fact that it is very unlikely that “similar” results in these calculations would mean “identical” results. Input PQR files for calculation of electrostatic potentials addressed in the next subsection were then constructed from structure PDB files with modified labelling of protonated residues using the Pdb2pqr 2.0^61,62^ service (available at http://nbcr-222.ucsd.edu/pdb2pqr_2.0.0/) without automatic pKa assignment. A PQR file is a modified PDB file in which occupancy and B-factor entries are replaced with atomic charges and radii, respectively^61^.

### Poisson-Boltzmann (PB) electrostatic potentials (EPs) and electric field

Using the PQR files mentioned in the preceding paragraph as input, PBEPs were computed by solving the nonlinear PB equation with the APBS 1.5^63^ program implemented as plug-in in PyMOL 2.3.2. Sequential focusing multigrid calculations in 3D grids typically composed of 129^3^ = 2,146,689 points (step size ~0.5 Å) at a 0.150 M NaCl concentration were performed with dielectric constants of 4 for proteins and 78.54 for water. The numerical output of PBEPs, *V*(**r**), was saved in OpenDX scalar format for further mapping onto molecular surfaces and calculation of 3D isopotential surfaces. These DX files were also used to compute the electric field ***E*** = −∇*V*(**r**) with VMD 1.9.3^49^. PBEP *V*(**r**) values are given in units of *kT*/*e*, with *k* being Boltzmann’s constant, *T* being the absolute temperature of 310 K, and *e* being the unit electron charge.

### Docking calculations to obtain the initial structures of the CD1d complexes for MD simulations

In the case of CD1d-lipid antigen complexes, protein-ligand docking calculations in blind mode were performed with AutoDock Vina 1.1.2^64^ and Chimera 1.13^51^. In all cases, the best solution (i.e., that having the lowest protein-ligand affinity DG computed with Vina’s scoring function^64^) matching similar geometries of lipid ligands after several rounds of docking calculations was selected for further MD study.

The following protein-protein docking methods were initially used in blind docking mode to prepare starting geometries of the CD1d-protein complexes: ClusPro 2.0^65^ (https://cluspro.bu.edu/), ZDOCK^66^ (http://zdock.umassmed.edu/) and pyDock^67^ (https://life.bsc.es/pid/pydockweb/). For CD1d-GM2AP complexes, only 2 models among the 30 models set by the 10 top solutions of ClusPro, ZDOCK and pyDock displayed proper orientation between the lipid-binding cavity of GM2AP and the portal region in CD1d. These 2 models were then used as initial input for refined protein-protein docking from user-defined binding segments with ZDOCK^66^ in contacting-residues mode and HADDOCK 2.2^68^ (http://milou.science.uu.nl/services/HADDOCK2.2/), which restricts the posing search by asking the user for a list of potential interacting residues. These refined solutions were then optimised with the RosettaDock protocol^69^ by selecting the highest-ranked solution among the top 10 best-scored poses.

For CD1d-SapA dimer complexes, the geometry of the human SapA dimer was first modelled with Chimera 1.13^51^ by structural superposition with the geometry of the murine SapA dimer in the recently reported crystal structure of its complex with the GALC enzyme (PDB id 5NXB)^42^. The number of blind-docking solutions for the CD1d-SapA dimer complex with the proper orientation between the opening of the inner cavity of the SapA dimer and the portal region of CD1d were the following: 5 out of 10 top models in ClusPro, 6 out of 10 top models in ZDOCK, and 8 out of 10 top models in pyDock. The 4 best positioned solutions of all 19 models were then refined with RosettaDock as was done with the CD1d-GM2AP complex above. These four geometries supplied by RosettaDock were then used in 20 ns MD exploratory simulations to finally select the best structure for further MD study.

In the case of the CD1d-Pru p 3 complexes, the number of blind docking solutions with proper positioning of the allergen with respect to the portal region of CD1d were the following: 9 out of 10 top models in ClusPro, 10 out of 10 top models in ZDOCK, and 8 out of 10 top models in pyDock. In addition, an initial geometry of Pru p 3 located at a large distance of the portal region of CD1d was also used as a RosettaDock input in order to explore the orientation of the hydrophobic tunnel of the allergen with respect to the CD1d portal. The 4 highest-ranked solutions among their top 10 best-scored poses were finally selected as initial geometries for further MD study.

### All-atom molecular dynamics (MD) simulations

Except where noted in the main text, all-atom MD simulations were run over simulation times of 100 ns with the protonation states obtained as explained above and the CHARMM 3.6 force field for proteins^70^. MD calculations were performed with the high-performance computing Power-MPI version of NAMD 1.12^71^ in the Magerit3 supercomputer of Technical University of Madrid. All protein-ligand, protein-protein and protein-protein-ligand systems were prepared (and ligands parametrised) for their MD study using the PDB Reader service of CHARMM-GUI^72^ (http://www.charmm-gui.org/). Periodic solvation boxes were constructed with 15 Å spacing and water molecules according to the TIP3P model^73^. Sodium and chloride ions were added to counter the total charges of the protein systems setting a 0.150M salt concentration. The particle-mesh Ewald summation method was used for long-range electrostatics and a 10 Å cutoff was set for short-range non-bonded interactions. Initial geometries were first minimised at 5,000 conjugate-gradient steps, water was then equilibrated at 298 K and 1 atm for 100 ps at 2 fs time steps, and production runs were then performed for 100 ns at 2 fs time steps (50 million steps per calculation) in the NPT ensemble at 1 atm and 298 K. Langevin dynamics for T control and the Nosé-Hoover Langevin piston method for P control were employed. NAMD output was stored every 20,000 steps, giving trajectories composed of 2,500 frames that were processed and analysed with VMD 1.9.3^49^ and Carma 1.7^55^.

## Supplementary information

Supplementary Information.

## References

[1] Rossjohn J (2015). T Cell Antigen Receptor Recognition of Antigen-Presenting Molecules. Annu. Rev. Immunol..

[2] Adams EJ, Luoma AM (2013). The Adaptable Major Histocompatibility Complex (MHC) Fold: Structure and Function of Nonclassical and MHC Class I–Like Molecules. Annu. Rev. Immunol..

[3] Josefowicz SZ, Lu L-F, Rudensky AY (2012). Regulatory T Cells: Mechanisms of Differentiation and Function. Annu. Rev. Immunol..

[4] Neefjes J, Jongsma MLM, Paul P, Bakke O (2011). Towards a systems understanding of MHC class I and MHC class II antigen presentation. Nat. Rev. Immunol..

[5] Barral DC, Brenner MB (2007). CD1 antigen presentation: How it works. Nat. Rev. Immunol..

[6] Vartabedian VF, Savage PB, Teyton L (2016). The processing and presentation of lipids and glycolipids to the immune system. Immunol. Rev..

[7] Moody DB, Cotton RN (2017). Four pathways of CD1 antigen presentation to T cells. Curr. Opin. Immunol..

[8] Chancellor A, Gadola SD, Mansour S (2018). The versatility of the CD1 lipid antigen presentation pathway. Immunology.

[9] Calabi F, Jarvis JM, Martin L, Milstein C (1989). Two classes of CD1 genes. Eur. J. Immunol..

[10] Garcia-Alles LF (2011). Crystal structure of human CD1e reveals a groove suited for lipid-exchange processes. Proc. Natl. Acad. Sci..

[11] Birkinshaw RW (2015). αβ T cell antigen receptor recognition of CD1a presenting self lipid ligands. Nat. Immunol..

[12] Shahine, A. *et al*. A molecular basis of human T cell receptor autoreactivity toward self-phospholipids. *Sci. Immunol*. **2**, eaao1384/1–12 (2017). 10.1126/sciimmunol.aao1384 PMC6649662 29054999

[13] Mansour S (2016). Cholesteryl esters stabilize human CD1c conformations for recognition by self-reactive T cells. Proc. Natl. Acad. Sci..

[14] Brennan PJ, Brigl M, Brenner MB (2013). Invariant natural killer T cells: An innate activation scheme linked to diverse effector functions. Nat. Rev. Immunol..

[15] Wun KS (2011). A molecular basis for the exquisite CD1d-restricted antigen specificity and functional responses of natural killer T cells. Immunity.

[16] Brennan PJ (2017). Structural determination of lipid antigens captured at the CD1d–T-cell receptor interface. Proc. Natl. Acad. Sci..

[17] Florence WC, Bhat RK, Joyce S (2008). CD1d-restricted glycolipid antigens: Presentation principles, recognition logic and functional consequences. Expert Rev. Mol. Med..

[18] Biterova EI (2019). The crystal structure of human microsomal triglyceride transfer protein. Proc. Natl. Acad. Sci. USA.

[19] Teyton L (2018). Role of lipid transfer proteins in loading CD1 antigen-presenting molecules. J. Lipid Res..

[20] Zhou D (2004). Editing of CD1d-Bound Lipid Antigens by Endosomal Lipid Transfer Proteins. Science..

[21] Dong G, Wearsch PA, Peaper DR, Cresswell P, Reinisch KM (2009). Insights into MHC Class I Peptide Loading from the Structure of the Tapasin-ERp57 Thiol Oxidoreductase Heterodimer. Immunity.

[22] Blees A (2017). Structure of the human MHC-I peptide-loading complex. Nature.

[23] Fisette O, Wingbermühle S, Tampé R, Schäfer LV (2016). Molecular mechanism of peptide editing in the tapasin-MHC I complex. Sci. Rep..

[24] Nicholson MJ (2006). Small Molecules That Enhance the Catalytic Efficiency of HLA-DM. J. Immunol..

[25] Guce AI (2013). HLA-DO acts as a substrate mimic to inhibit HLA-DM by a competitive mechanism. Nat. Struct. Mol. Biol..

[26] Garzón D, Anselmi C, Bond PJ, Faraldo-Gómez JD (2013). Dynamics of the Antigen-binding Grooves in CD1 Proteins. J. Biol. Chem..

[27] López-Sagaseta J, Sibener LV, Kung JE, Gumperz J, Adams EJ (2012). Lysophospholipid presentation by CD1d and recognition by a human Natural Killer T-cell receptor. EMBO J..

[28] Cubells-Baeza N (2017). Identification of the ligand of Pru p 3, a peach LTP. Plant Mol. Biol..

[29] Van Ree R (2002). Clinical importance of non-specific lipid transfer proteins as food allergens. Biochemical Society Transactions.

[30] Salcedo G, Sánchez-Monge R, Barber D, Díaz-Perales A (2007). Plant non-specific lipid transfer proteins: An interface between plant defence and human allergy. Biochimica et Biophysica Acta - Molecular and Cell Biology of Lipids.

[31] Tordesillas L (2017). Mechanisms underlying induction of allergic sensitization by Pru p 3. Clin. Exp. Allergy.

[32] Hung JT, Huang JR, Yu AL (2017). Tailored design of NKT-stimulatory glycolipids for polarization of immune responses. J. Biomed. Sci..

[33] Koch M (2005). The crystal structure of human CD1d with and without α-galactosylceramide. Nat. Immunol..

[34] Bauer JA, Pavlovíc J, Bauerová-Hlinková V (2019). Normal mode analysis as a routine part of a structural investigation. Molecules.

[35] Alexandrov V (2005). Normal modes for predicting protein motions: A comprehensive database assessment and associated Web tool. Protein Sci..

[36] Dangerfield EM (2012). Species-Specific Activity of Glycolipid Ligands for Invariant NKT Cells. ChemBioChem.

[37] Wun KS (2018). T cell autoreactivity directed toward CD1c itself rather than toward carried self lipids. Nat. Immunol..

[38] Zeng ZH (1997). Crystal structure of mouse CD1: An MHC-like fold with a large hydrophobic binding groove. Science..

[39] Wright CS, Mi LZ, Lee S, Rastinejad F (2005). Crystal structure analysis of phosphatidylcholine - GM2-activator product complexes: Evidence for hydrolase activity. Biochemistry.

[40] Popovic K, Holyoake J, Pomes R, Prive GG (2012). Structure of saposin A lipoprotein discs. Proc. Natl. Acad. Sci..

[41] Garrido-Arandia M, Cuevas-Zuviría B, Díaz-Perales A, Pacios LF (2018). A comparative study of human saposins. Molecules.

[42] Hill CH (2018). The mechanism of glycosphingolipid degradation revealed by a GALC-SapA complex structure. Nat. Commun..

[43] Cuevas-Zuviría B, Garrido-Arandia M, Díaz-Perales A, Pacios LF (2019). Energy landscapes of ligand motion inside the Tunnel-like cavity of lipid transfer proteins: The case of the Pru p 3 allergen. Int. J. Mol. Sci..

[44] Wong LH, Gatta AT, Levine TP (2019). Lipid transfer proteins: the lipid commute via shuttles, bridges and tubes. Nat. Rev. Mol. Cell Biol..

[45] Borbulevych OY (2009). T Cell Receptor Cross-reactivity Directed by Antigen-Dependent Tuning of Peptide-MHC Molecular Flexibility. Immunity.

[46] Pasquato N (2006). Crystal structure of peach Pru p 3, the prototypic member of the family of plant non-specific lipid transfer protein pan-allergens. J. Mol. Biol..

[47] Holm L, Laakso LM (2016). Dali server update. Nucleic Acids Res..

[48] Holm L, Park J (2000). DaliLite workbench for protein structure comparison. Bioinformatics.

[49] Humphrey W, Dalke A, Schulten K (1996). VMD: Visual molecular dynamics. J. Mol. Graph..

[50] Goddard Thomas D (2017). UCSF ChimeraX: Meeting modern challenges in visualization and analysis. Protein Sci..

[51] Pettersen EF (2004). UCSF Chimera - A visualization system for exploratory research and analysis. J. Comput. Chem..

[52] Li H, Chang YY, Lee JY, Bahar I, Yang LW (2017). DynOmics: Dynamics of structural proteome and beyond. Nucleic Acids Res..

[53] Volkamer A, Kuhn D, Grombacher T, Rippmann F, Rarey M (2012). Combining global and local measures for structure-based druggability predictions. J. Chem. Inf. Model..

[54] Craig IR, Pfleger C, Gohlke H, Essex JW, Spiegel K (2011). Pocket-space maps to identify novel binding-site conformations in proteins. J. Chem. Inf. Model..

[55] Koukos PI, Glykos NM (2013). Grcarma: A fully automated task-oriented interface for the analysis of molecular dynamics trajectories. J. Comput. Chem..

[56] Olsson MHM, SØndergaard CR, Rostkowski M, Jensen JH (2011). PROPKA3: Consistent treatment of internal and surface residues in empirical p K a predictions. J. Chem. Theory Comput..

[57] Søndergaard CR, Olsson MHM, Rostkowski M, Jensen JH (2011). Improved treatment of ligands and coupling effects in empirical calculation and rationalization of p K a values. J. Chem. Theory Comput..

[58] Anandakrishnan R, Aguilar B, Onufriev AV (2012). H++ 3.0: Automating pK prediction and the preparation of biomolecular structures for atomistic molecular modeling and simulations. Nucleic Acids Res..

[59] Myers J, Grothaus G, Narayanan S, Onufriev A (2006). A simple clustering algorithm can be accurate enough for use in calculations of pKs in macromolecules. Proteins Struct. Funct. Genet..

[60] Kilambi KP, Gray JJ (2012). Rapid calculation of protein pKa values using rosetta. Biophys. J..

[61] Dolinsky TJ, Nielsen JE, McCammon JA, Baker NA (2004). PDB2PQR: An automated pipeline for the setup of Poisson-Boltzmann electrostatics calculations. Nucleic Acids Res..

[62] Dolinsky TJ (2007). PDB2PQR: Expanding and upgrading automated preparation of biomolecular structures for molecular simulations. Nucleic Acids Res..

[63] Jurrus E (2018). Improvements to the APBS biomolecular solvation software suite. Protein Sci..

[64] Trott O, Olson A (2010). Autodock vina: improving the speed and accuracy of docking. J. Comput. Chem..

[65] Kozakov D (2017). The ClusPro web server for protein-protein docking. Nat. Protoc..

[66] Pierce BG (2014). ZDOCK server: Interactive docking prediction of protein-protein complexes and symmetric multimers. Bioinformatics.

[67] Jiménez-García B, Pons C, Fernández-Recio J (2013). pyDockWEB: A web server for rigid-body protein-protein docking using electrostatics and desolvation scoring. Bioinformatics.

[68] Van Zundert GCP (2016). The HADDOCK2.2 Web Server: User-Friendly Integrative Modeling of Biomolecular Complexes. J. Mol. Biol..

[69] Lyskov S, Gray JJ (2008). The RosettaDock server for local protein-protein docking. Nucleic Acids Res..

[70] Best RB (2012). Optimization of the additive CHARMM all-atom protein force field targeting improved sampling of the backbone φ, ψ and side-chain χ1 and χ2 Dihedral Angles. J. Chem. Theory Comput..

[71] Phillips JC (2005). Scalable molecular dynamics with NAMD. J. Comput. Chem..

[72] Jo S (2014). CHARMM-GUI PDB manipulator for advanced modeling and simulations of proteins containing nonstandard residues. Adv. Protein Chem. Struct. Biol..

[73] Jorgensen WL, Chandrasekhar J, Madura JD, Impey RW, Klein ML (1983). Comparison of simple potential functions for simulating liquid water. J. Chem. Phys..

