# Supplementary material for: Dynamic plasticity of the lipid antigen-binding site of CD1d is crucially favoured by acidic pH and helper proteins
Source: Sci Rep. 2020 Mar 31;10:5714. doi: 10.1038/s41598-020-62833-y (PMC7109084; doi:10.1038/s41598-020-62833-y)
Supplement: Supplementary file 1 — Supplementary Information. [file 41598_2020_62833_MOESM1_ESM.pdf]

Dynamic plasticity of the lipid antigen-binding site of CD1d is crucially favoured by acidic pH and helper proteins

Bruno Cuevas-Zuviría, Marina Mínguez-Toral, Araceli Díaz-Perales,
María Garrido-Arandia & Luis F. Pacios*

Supplementary Information

	Page
Supplementary Figure 1	S2
Supplementary Figure 2	S3
Supplementary Figure 3	S4
Supplementary Figure 4	S5
Supplementary Figure 5	S6

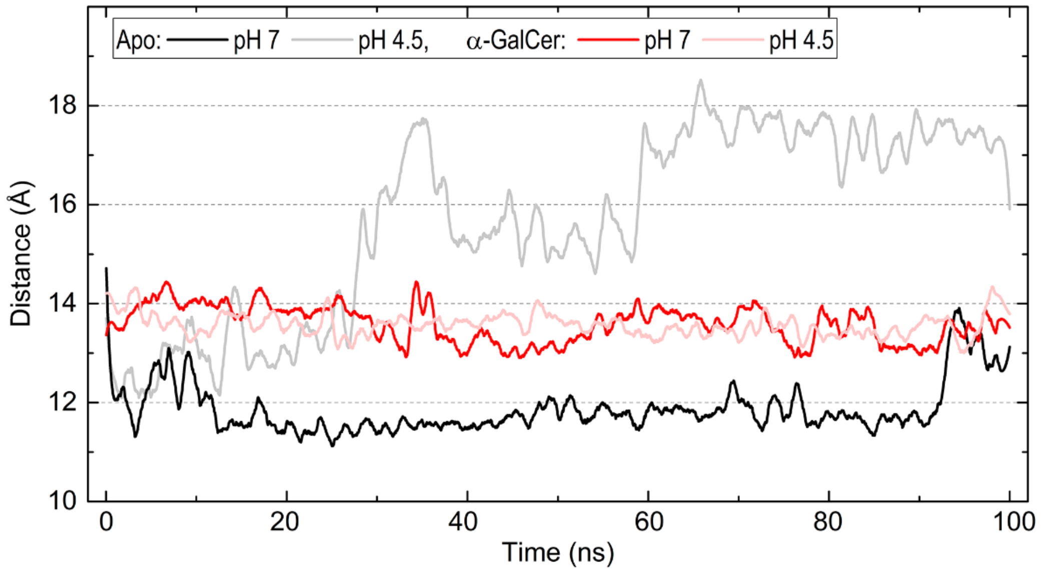

Supplementary Figure 1. Change along 100 ns all-atom MD simulations of the Cα.F77-Cα.D151 distance in the apo-form of CD1d (lipid-unbound chain in 1ZT4) and in the complex with α-GalCer (lipid-bound chain) at pH 7 and 4.5 (plots smoothed with Savitzky-Golay filtering)

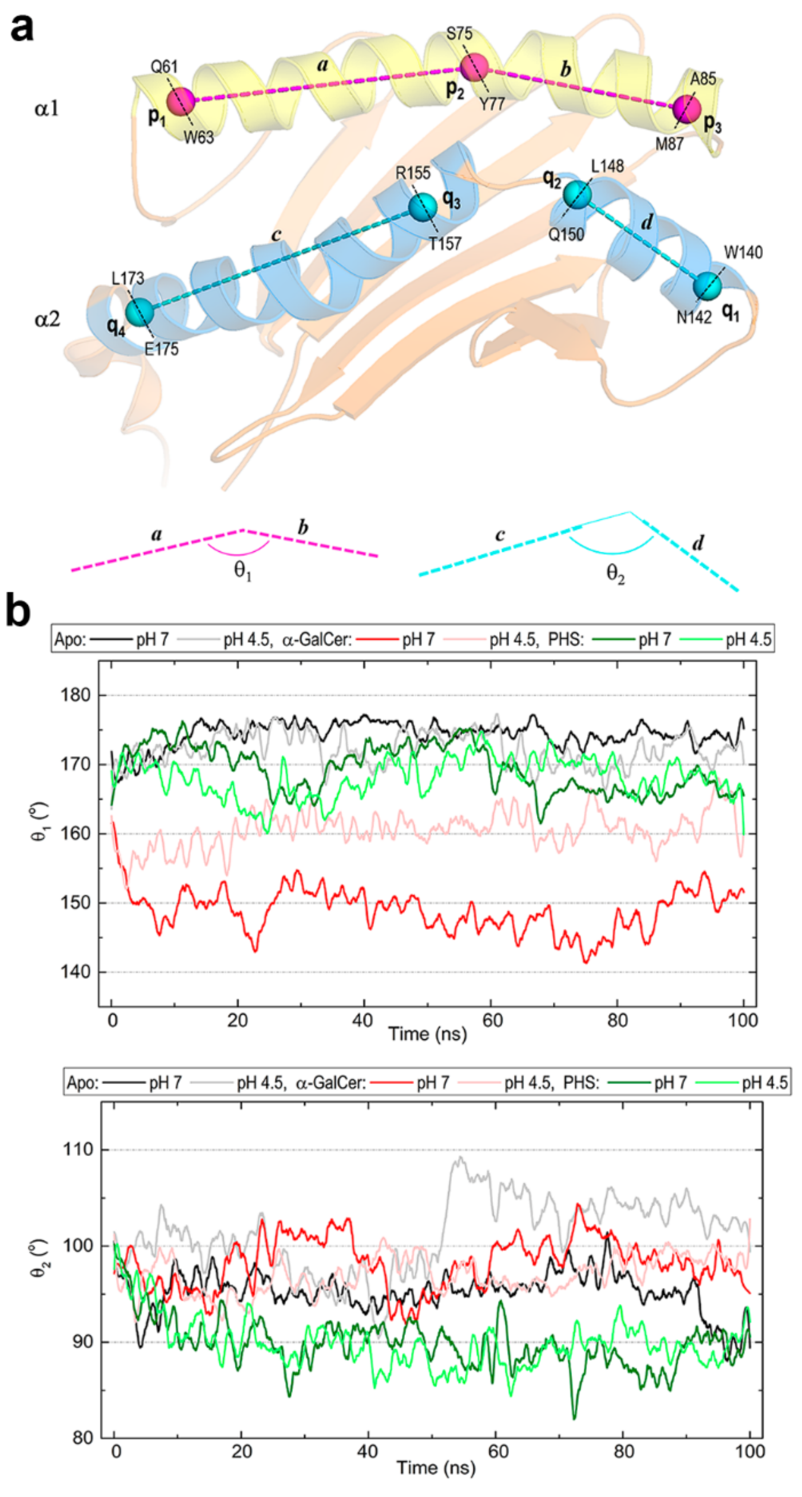

Supplementary Figure 2. Change along 100 ns all-atom MD simulations of the angles measuring the bending of helices α1 and α2 in CD1d. a Definition of the angles θ_1 and θ_2 in helices α1 and α2, respectively, measured as the angles between vectors ***a*** and ***b*** (θ_1) and between vectors ***c*** and ***d*** (θ_2). These four vectors are defined by the middle points of the distances between alpha carbons of residues labelled at points $\mathbf{p_1}$, $\mathbf{p_2}$, $\mathbf{p_3}$ (θ_1) and $\mathbf{q_1}$, $\mathbf{q_2}$, $\mathbf{q_3}$, $\mathbf{q_4}$ (θ_2). **b** Variation of the angles θ_1 and θ_2 in the apo-form of CD1d and in its complexes with α-GalCer and PHS at pH 7 and pH 4.5 (plots smoothed with Savitzky-Golay filtering).

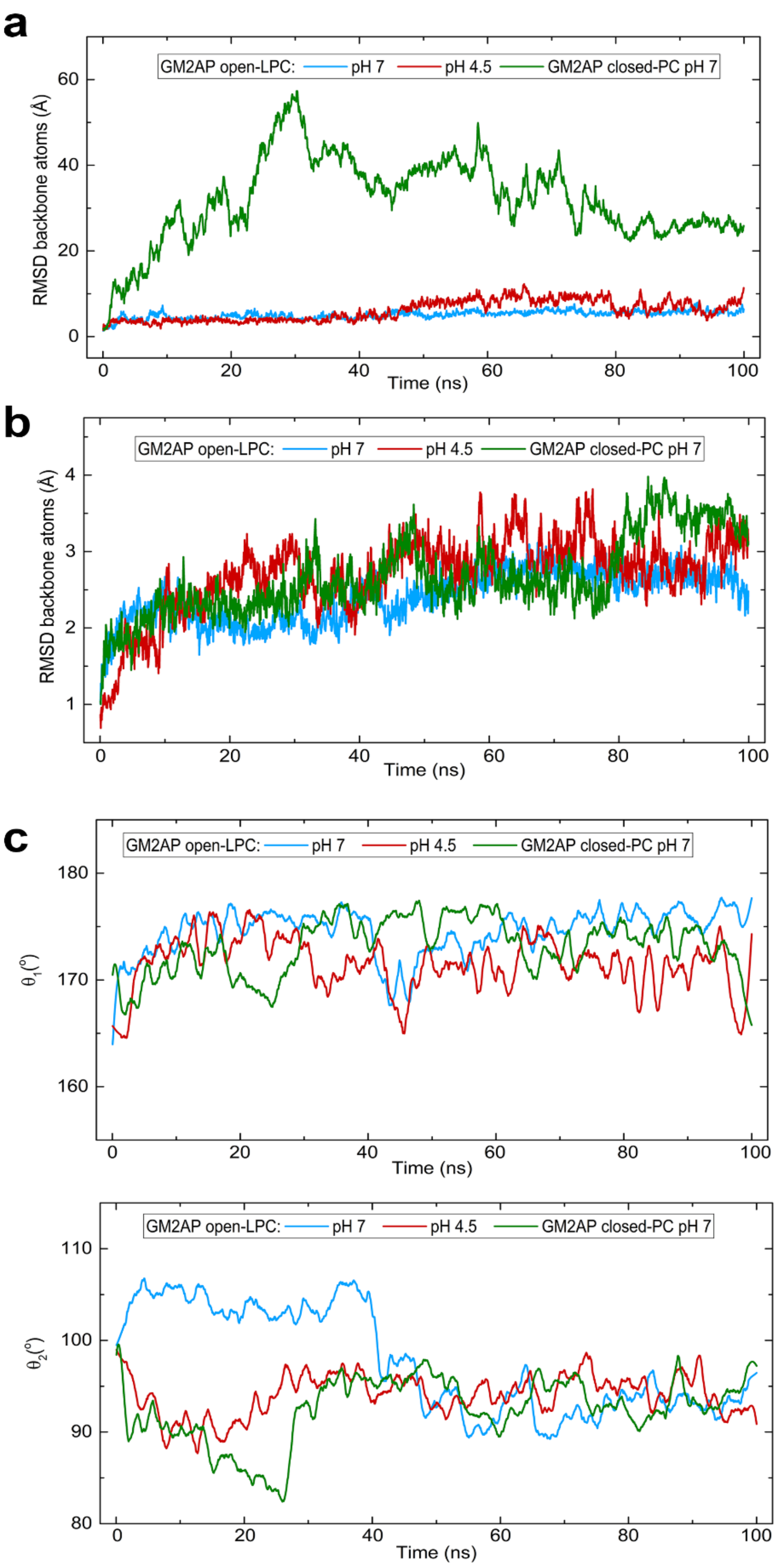

Supplementary Figure 3. Results of 100 ns all-atom MD simulations of CD1d-GM2AP (open)-OLA-LPC and CD1d-GM2AP (closed)-PC complexes. a. RMSD computed with backbone atoms of both proteins. The complex with closed GM2AP dissociates at the very beginning of the simulation which explains the large increase of its RMSD curve. **b.** RMSD computed with backbone atoms of CD1d. **c.** Variation of the angles θ_1 and θ_2 defined in Supplementary Figure 2 measuring the bending of the helices $\alpha 1$ and $\alpha 2$ in CD1d, respectively.

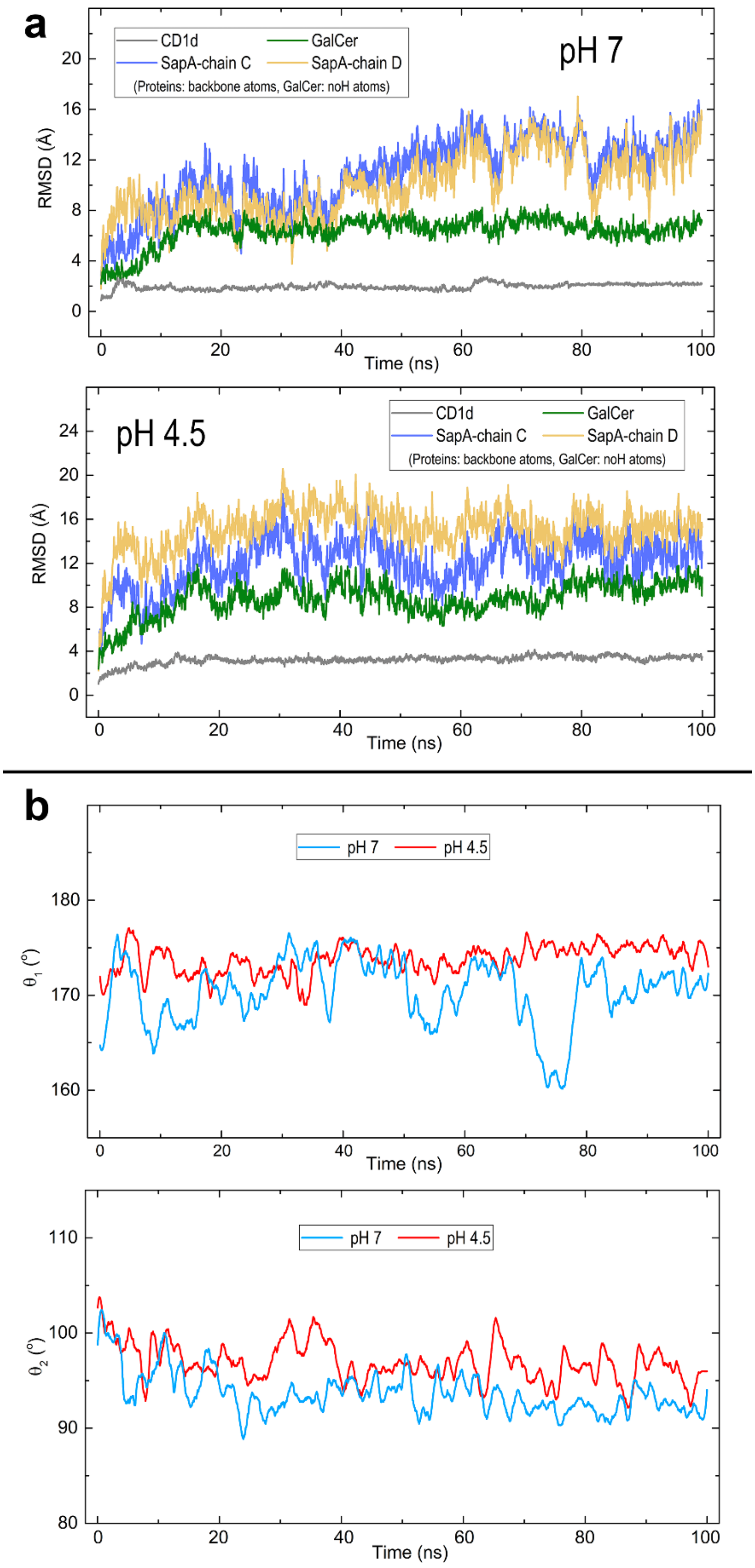

Supplementary Figure 4. Results of 100 ns all-atom MD simulations of the CD1d-SapA (open) dimer-α-GalCer complex at pH 7 and pH 4.5. a. RMSD of protein chains (computed with backbone atoms) and of α-GalCer (computed with noH atoms). **b.** Variation of the angles θ_1 and θ_2 defined in Supplementary Figure 2 measuring the bending of the helices α1 and α2 in CD1d, respectively.

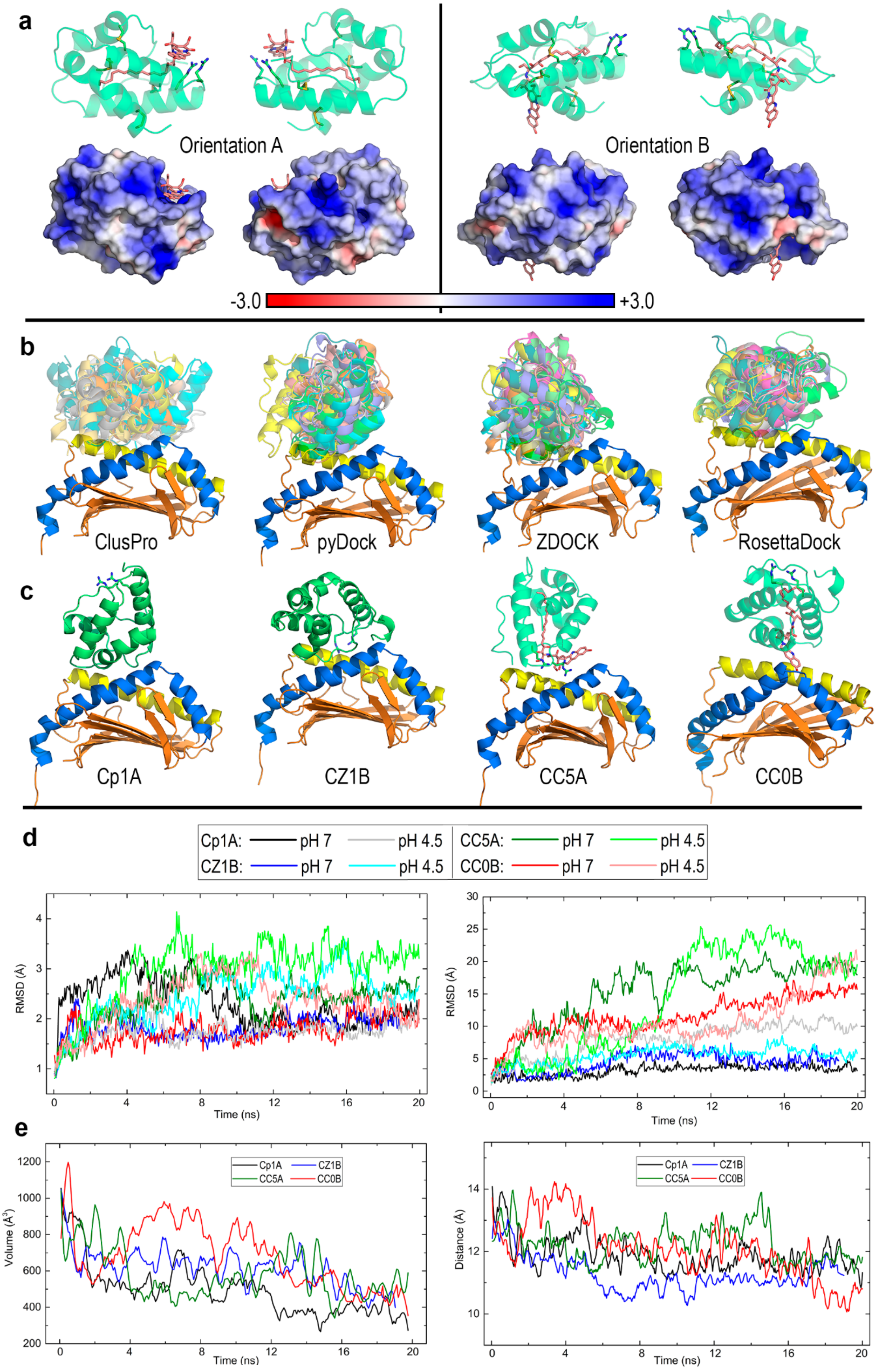

Supplementary Figure 5. The major peach allergen Pru p 3 and CD1d. a. Final structures of 100 ns MD simulations of Pru p 3 in complex with its lipid ligand (sticks with carbons in salmon) in the

two orientations A and B inside the hydrophobic tunnel-like cavity of the allergen (Ref. 34 in the main text). Two arginine residues at one end of the tunnel are depicted for reference. The right view is obtained from the left view upon a 180° rotation around a vertical axis in both orientations. The PBEP mapped onto the protein surface is shown for all the structures displayed in the top row. **b.** Geometries of the CD1d-Pru p 3 complex with proper location of the allergen on top of the CD1d portal obtained among the 10 best solutions of protein-protein blind docking calculations with the four methods indicated. The number of these geometries drawn in the figure is: 9 in ClusPro, 8 in pyDock, 10 in ZDOCK, and 10 in RosettaDock. **c.** Initial structures selected for exploratory 20 ns MD simulations of the CD1d-Pru p 3 complex in the absence (Cp1A and CZ1B) and in the presence (CC5A and CC0B) of the ligand of Pru p 3. The last letter in these four labels refer to the relative orientation of Pru p 3 with respect to CD1d (the same arginine residues depicted in **a.** are also shown for reference). **d.** RMSD of both proteins computed with backbone atoms in the complexes indicated in **c.** obtained in the exploratory MD simulations at pH 7 and pH 4.5. **e.** Change along these exploratory MD simulations of the cavity volumes (left panel) and groove portal distance (right panel) in CD1d at pH 7 (plots at pH 4.5 are rather similar and have been omitted).
